# Dynamic assessment of signal entropy for prognostication and secondary brain insult detection after traumatic brain injury

**DOI:** 10.1186/s13054-024-05228-z

**Published:** 2024-12-30

**Authors:** Stefan Yu Bögli, Ihsane Olakorede, Erta Beqiri, Xuhang Chen, Ari Ercole, Peter Hutchinson, Peter Smielewski

**Affiliations:** 1 Brain Physics Laboratory, Division of Neurosurgery, Department of Clinical Neurosciences, University of Cambridge, Cambridge, UK; 2 Division of Neurosurgery, Department of Clinical Neurosciences, University of Cambridge, Cambridge, UK; 3 Division of Anaesthesia, Department of Medicine, University of Cambridge, Cambridge, UK; 4 Department of Neurology and Neurocritical Care Unit, Clinical Neuroscience Center, University Hospital Zurich, University of Zurich, Zurich, Switzerland

**Keywords:** Entropy, Complexity, Traumatic brain injury, Multimodality monitoring

## Abstract

**Background:**

Entropy quantifies the level of disorder within a system. Low entropy reflects increased rigidity of homeostatic feedback systems possibly reflecting failure of protective physiological mechanisms like cerebral autoregulation. In traumatic brain injury (TBI), low entropy of heart rate and intracranial pressure (ICP) predict unfavorable outcome. Based on the hypothesis that entropy is a dynamically changing process, we explored the origin and value of entropy time trends.

**Methods:**

232 continuous recordings of arterial blood pressure and ICP of TBI patients with available clinical information and 6-month outcome (Glasgow Outcome Scale) were accessed form the Brain Physics database. Biosignal entropy was estimated as multiscale entropy (MSE) that aggregates entropy at several time scales (20 coarse graining steps starting from 0.1 Hz). MSE was calculated repeatedly for consecutive, overlapping 6 h segments. Percentage monitoring time (ptime) or dosage (duration*level/hour) below different cutoffs were evaluated against outcome using univariable and multivariable analyses, and propensity score matching. Associations to clinical and monitoring metrics were explored using correlation coefficients. Lastly, individual secondary brain insults (deviations in ICP, cerebral perfusion pressure – CPP, or pressure reactivity) were assessed in relation to changes in MSE.

**Results:**

Increased MSE abp and MSE cpp ptime (OR 1.28 (1.07–1.58) and OR 1.50 (1.16–2.03) for MSE abp and cpp respectively) and dose (OR 1.12 (1.02–1.27) and OR 1.21 (1.06–1.46) for MSE abp and cpp respectively) were associated with poor outcome even after propensity score matching within multivariable models correcting for ICP, CPP, and the pressure reactivity index. MSE trajectories differed significantly dependent on outcome. The entropy metrics displayed weak correlations to clinical parameters. Individual episodes of deranged physiology were associated with decreases in the MSE metrics from both cerebral and systemic biosignals.

**Conclusions:**

Biosignal entropy of changes dynamically after TBI. The assessment of these variations augments individualized, dynamic, outcome prognostication and identification of secondary cerebral insults. Additionally, these explorations allow for further exploitation of the extensive physiological data lakes acquired for each TBI patient within an intensive care environment.

**Supplementary Information:**

The online version contains supplementary material available at 10.1186/s13054-024-05228-z.

## Introduction

Traumatic brain injury (TBI) leads to secondary local and systemic effects above and beyond the disruption of brain function caused by the primary injury itself. The clinical course and overall trajectories after TBI can vary largely and at times rapidly between and within patients [1]. Secondary deterioration after TBI is common both due to secondary brain injury as well as the often-coexisting extracranial injury [2]. To sustain internal homeostasis, various interacting physiologic systems must counteract these disruptions leading to complex patterns of variations of measured physical quantities, such as pressures or flows, across different temporal and spatial scales [3]. These patterns are captured by different biosignals (defined as any physiological signal that can be continually measured or monitored such as arterial blood pressure – ABP, or heart rate—HR). Entropy quantifies such complex variability and correspondingly the level of disorder within a system. Multiscale entropy (MSE) in particular has been shown to be a particularly suitable metric since it estimates sample entropy across a range of increasingly downsampled (i.e. averaged) data [3, 4]. This method of coarse-graining, in effect, allows for the evaluation of changes in entropy across different time scales and is more resistant to noise compared to metrics of entropy evaluated using a single scale.

Loss of such complex patterns of variation, termed “decomplexification”, has been associated with unfavorable outcome or mortality in various diseases including TBI [5, 6]. From a physiological perspective low MSE is believed to reflect failure of different regulatory mechanisms possibly reflecting a more rigid cardio/cerebrovascular system. Based on the vast number of studies on heart rate variability, the first explorations of MSE in trauma were introduced using HR as the biosignal of interest. In the overall trauma population low MSE of HR was associated with mortality when assessed within the first 24 h [7]. Interestingly, similar results were found for patients with isolated head injury, isolated torso injury, and polytrauma patients. Sample entropy of a single scale, acquired in the resuscitation bay in trauma patients, associated with the subsequent need for life saving interventions in spite of otherwise normal vital signs [8]. Sample entropy of HR was also closely linked to the injury severity score as well as the subsequent length of stay in the intensive care unit [9]. In TBI, additional biosignals were explored with MSE of intracranial pressure (ICP) being associated with outcome and additionally intracranial hypertension and plateau waves [10].

Within the last decades, rather than using absolute or average values of biosignals, the evaluation of the extent (i.e. duration or dose) outside the suggested ranges has augmented the understanding of physiology taking into account an innate resistance to short durations of high or long durations of very low derangements [11, 12]. To date, in TBI, MSE has only been evaluated as a single value per patient. Physiological regulatory systems, however, are innately non-stationary, having to adjust for changing factors such as disease progression. Our primary aim was to evaluate entropy dose or the percentage of monitoring time (ptime) below specific cutoffs after TBI in relation to patient outcomes. We hypothesized that increased MSE dose or prolonged duration spent below these cutoffs would be associated with worse outcomes following TBI. Secondary aims included exploring potential associations between MSE and physiological metrics, either overall or during specific physiological insults.

## Materials and methods

The acquisition and use of this dataset, which exclusively comprises routinely collected data, was approved by the local ethics committee (REC 23/YH/0085). The dataset includes high-resolution monitoring data and clinical descriptors from consecutive TBI patients admitted to the Neurocritical Care Unit at Addenbrooke’s Hospital, Cambridge University Hospital NHS Foundation Trust, University of Cambridge. Data acquisition occurs continuously while the patient remains in the NCCU and respective monitoring is deemed necessary. If care is redirected—for example, to palliative measures—monitoring is discontinued. For all other cases, monitoring continues for the entirety of the patient's stay in the unit. This approach ensures comprehensive data collection during active treatment. All data were obtained as part of routine care, encompassing invasive neuromonitoring and regular physiological monitoring. No additional study-specific data were collected, and therefore, the requirement for informed consent was waived.

### Study population

Patients admitted between 03.2021 and 12.2023 were evaluated for inclusion. Inclusion criteria were: 1. Acute TBI with invasive ICP monitoring; 2. Available 6-month outcome (Glasgow Outcome Scale – GOS). The exclusion criteria were: 1. No monitoring data; 2. loss of follow up with missing 6-month outcome; 3. Incomplete clinical descriptors. Patients were treated based on previously described protocols [13, 14] and in accordance with the guidelines by the Brain Trauma Foundation [15].

### Data acquisition

High resolution physiological data (250 Hz) was collected in real time at the bedside using the ICM + software running on a laptop at the bedside (ICM + software ®, Cambridge Enterprises, University of Cambridge, UK). ICP was measured using intraparenchymal wires (Codman ICP MicroSensor, Codman & Shurtleff, Raynham, Massachusetts). ABP was measured using arterial lines (Baxter Healthcare, Deerfield, Illinois) inserted to the radial or femoral artery and zeroed at the level of the foramen of Monroe. The following clinical data was extracted from the database: sex, age, Glasgow Coma Scale (GCS), pupillary reactivity (both reactive vs. one reactive vs. none reactive), presence of intracranial bleeding (extradural hematoma—EDH, intracerebral hematoma—ICH, subdural hematoma—SDH, contusion, traumatic subarachnoid hemorrhage—SAH), presence of extracerebral injuries (divided into injuries to thorax, abdomen, extremities, pelvic, skull, or spine), presence of isolated TBI (no extracranial injuries), decompressive craniectomy (DC), and GOS. GOS was assessed at 6 months after ictus during outpatient consultations or via telephone interviews by trained staff. Outcome was evaluated assessing the ordinal scale (good recovery vs. moderate disability vs. severe disability vs. dead/vegetative) or as dichotomized outcome (GOS 1–4 vs. 5–8) or dead/vegetative vs. other outcome categories (GOS 1/2 vs. 3–8).

### Data preprocessing

The high-resolution (i.e. waveform) monitoring data was preprocessed using ICM+. Raw ABP and ICP signals were curated to remove the following artifacts: 1. Sections with arterial line failure (continuous reduction of the arterial blood pressure amplitude followed by flushing) were removed manually; 2. Values of ABP below 0 or above 300 mmHg and sections with a pulse amplitude below 15 mmHg were removed automatically; 3. Values of ICP below -30 or above 200 mmHg, with low amplitude (< 0.04 mmHg) or with a 95% Spectral edge frequency above 10 Hz (high-frequency noise) were removed automatically. Based on the curated data, heart rate (HR – calculated by extracting the fundamental frequency of ABP within the limits of 40 to 180 beats per minute) and ICP amplitude (AMP – fundamental amplitude of ICP within the limits of 40 to 180 beats per minute) were estimated. The resulting artifact free data was then processed to acquire 10 s averages of ABP, ICP, AMP, CPP (difference between ABP and ICP), and HR. For ABP and ICP this coarse-graining removes the cardiac and respiratory components of the signal.

### Multiscale entropy analysis

MSE was calculated as previously described [4, 16]. Sample entropy [17] was calculated for a total of 20 steps (with 1 being the raw data and each consecutive step describing the number of consecutive samples to be averaged leading to coarse graining of the data) with a sequence length of m = 2 and a tolerance of 0.15. Sample entropy estimates whether corresponding sequences of length m remain the same when extending the sequence by one sample (i.e. m + 1). Its value is the negative natural logarithm of the ratio between the number of *m* + 1 length and corresponding m patterns. The resulting area under the curve (AUC) of the plotted sample entropies at all steps was termed MSE. Increasing MSE represents increasing entropy and thus complexity of the signal. To allow for the analysis of time-trends, MSE was calculated every 5 min for consecutive overlapping 6 h data segments with a minimum of 1000 valid samples. MSE was calculated for each of the biosignals resulting in the metrics MSE abp, MSE cpp, MSE hr, MSE icp, and MSE amp. In addition to the overall levels of MSE, percentage monitoring time (ptime) or dose (area below the curve) below specified MSE cutoffs [9, 12, 15] were calculated relative to the amount of available artefact free monitoring data. The thresholds were defined based on previous reports of MSE and based on an initial exploration of the distributions.

### Statistical analysis

Statistical analysis and figure preparation was performed in R Studio (R version 4.3.2—https://www.r-project.org/—packages used: *gtsummary, rstatix, MatchIt, cobalt, lme4, MASS, Hmisc, ggplot2*).

Descriptive variables are reported as counts (percentages) or median (interquartile range – IQR). Different statistical methods were explored to assess the association between MSE dose or ptime and outcome. Univariable, multivariable, and ordinal analyses were performed. A significance level of *p* < 0.05 was set, without adjustment for multiple testing due to the exploratory nature of the study and the different tests used for exploration.

Univariable tests: First, the different MSE variables were compared to outcome using Kruskal–Wallis tests assessing group level differences and then using Wilcoxon rank sum tests comparing between subgroup differences. The diagnostic performance of the metrics was assessed by plotting their receiver operating curves and extracting AUC, and the derived measures sensitivity, specificity, and accuracy (based on the Youden index).

Multivariable tests: Two multivariable approaches were used to assess the relevance of the MSE metrics when corrected for known predictors of outcome in TBI. First, we applied propensity score matching to account for covariates [18]. The propensity scores were estimated using logistic regression comparing favorable vs. unfavorable outcome. The following parameters were included to estimate the propensity scores: age, sex, motor GCS, pupillary reactivity, type of hemorrhage – EDH/ICH/SDH/contusion/SAH, isolated TBI vs. polytrauma, extracranial injury (to the abdomen, extremities, pelvis, skull, spine, or thorax seperately), and DC. Propensity score matching was performed using the nearest-neighbor method with a caliper of 0.2 and 1:1 matching to ensure that matches were within a reasonable distance in terms of their propensity scores. Quality of the matching procedure was verified visually (density and point distribution plots) and quantitatively (evaluation of absolute mean difference before and after matching and basic statistical analysis). The different MSE metrics were then fed into multivariable logistic regression models including other multimodal monitoring metrics (i.e. average ICP, CPP, PRx). Second, a sliding dichotomy approach [18] was applied. The sliding dichotomy approach creates a relative outcome scale for each patient. The definition of favorable and unfavorable outcome is adjusted depending on the propensity score (calculated as described above) for each patient. For patients with excellent prognosis at admission, only GOS 5 is considered favorable, while for patients with poor prognosis at admission, survival would already be regarded as a favorable outcome. Based on the propensity scores, the patients were divided into three groups of roughly equal size with low, intermediate, and high likelihood of unfavorable outcome. The definition of favorable and unfavorable outcome was then adjusted for each patient. In patients with low likelihood only GOS of 5 was considered favorable, while for the patients with intermediate likelihood GOS of 4–5 and for the patients with high likelihood GOS of 3–5 was considered favorable. The patients were then assessed using logistic regression based on this adjusted outcome definition. Additional secondary multivariable analyses were performed to further explore the value of the MSE metrics: 1. A second multivariable propensity score matching based approach was explored including ICP, CPP and PRx doses instead of averages to mimic the dynamic assessment of MSE explored in this analysis. For this purpose, patients were matched as described above and then compared using multivariable regression models including ICP, CPP and PRx doses (i.e. ICP dose above 20 mmHg, CPP dose below 60 mmHg, PRx dose above 0.3) and either MSE metric. 2. A backwards stepwise elimination regression was performed. In light of the moderate sample size with various known clinical and monitoring metrics associated with outcome, the backward stepwise elimination process was chosen since it allows for automated simplification of models (with many initially added variables), retaining only the key predictors of outcome. The initial model was built including the various clinical metrics (age, sex, motor GCS, pupillary reactivity, type of hemorrhage – EDH/ICH/SDH/contusion/SAH, isolated TBI vs. polytrauma, extracranial injury (to the abdomen, extremities, pelvis, skull, spine, or thorax separately), DC), the multimodality monitoring metrics (ICP, CPP, PRx) and either MSE metric. 3. To provide a quantification of improvement of the models when including the MSE metrics, we also explored the continuous net reclassification index [19]. The continuous net reclassification index represents the proportions of individuals correctly reclassified (true positive or true negative), minus the proportion misclassified when comparing new to old models. Considering the number of patients available the initial model included ICP dose above 20 mmHg, CPP dose below 60 mmHg, and PRx. This model was then compared to a model including the described metrics as well as one of the MSE metrics.

Time trends: Time trends were first explored visually exploring consecutive 6-h non-overlapping averages of the different MSE dose and ptime metrics. To adjust for possible non-linear changes, generalized additive models (GAM) were used to investigate the relationship between MSE metrics, day, and outcome. Formal statistical analysis was then performed using mixed effects model assessing the MSE metric trajectories depending on the fixed effects day and outcome including the patient as a random effect. Additionally, to explore whether differences were apparent early on, the entropy metrics were compared using the Kruskal–Wallis rank sum tests considering only the data acquired within the first 24 h after injury.

Correlations: To explore the possible physiological origin of the changes in MSE metrics, correlation coefficients between MSE metrics and different variables were estimated. For these estimations, we assessed the MSE as well as the physiology metrics considering the complete period available. Pearson correlation was used for continuous variables and Spearman correlation for ordinal metrics. The MSE metrics were correlated to the available clinical metrics described in the sections above. Additionally, the metrics were correlated to absolute mean HR, ABP, CPP, and ICP. Additionally, the following metrics were calculated and assessed: CPP dose and ptime below 60 mmHg, ICP dose and ptime above 20 mmHg. Cerebrovascular autoregulation was estimated using the proxy measure PRx [20] (absolute value as well as dose and ptime above 0.3) and its derivatives describing the optimal CPP (CPPopt) [21], and the lower limit of autoregulation (LLA) [22]. Lastly, individual episodes of CPP, ICP, or PRx insults were extracted and analyzed. Insults were defined as follows: a CPP insult was characterized by a decrease in CPP drop from above 60 mmHg for at least 15 min; an ICP insult was defined as an increase in ICP to above 20 mmHg for at least 15 min; and a PRx insult was identified by an increase in PRx to above 0.3 for at least 30 min [23] to allow for a sufficient change in biosignal state. MSE metrics, comparing the hour before and the hour after onset of each insult, were analyzed using paired t-tests and linear mixed-effects models. These models were adjusted for patient-specific random effects and included MSE and insult intensity (e.g., the change in CPP before vs. during the insult) as fixed effects. To further characterize the temporal relationship between MSE and the insults, cross-correlation analyses were performed (allowing a bidirectional lag of up to 30 min) to quantify the degree of similarity between the two timeseries as a function of a time lag, enabling the determination whether changes in MSE precede, coincide with, or follow the occurrence of changes in cerebral biosignals.

## Results

### Patient and monitoring characteristics

**Fig. 1:**
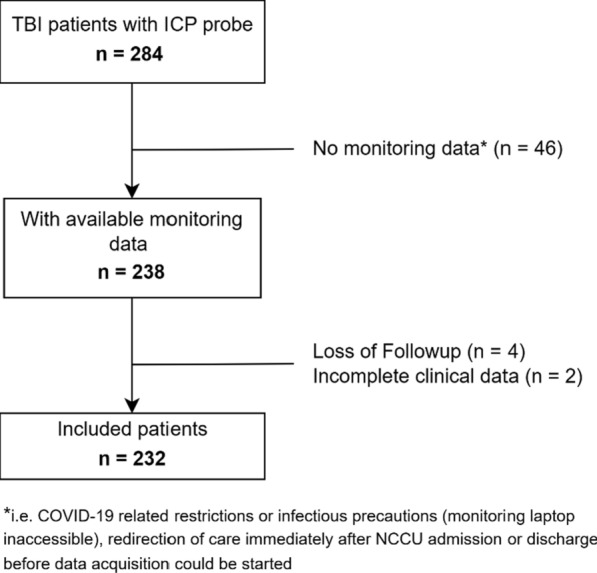
Inclusion/exclusion flowchart* *Abbreviations: ICP – Intracranial Pressure; TBI – Traumatic Brain Injury; NCCU – Neurocritical Care Unit

A total of 232 moderate-to-severe TBI patients with neuromonitoring and available 6-month GOS were evaluated. An inclusion exclusion flowchart is shown in Fig. 1. The clinical descriptors can be found in Table 1. At 6 months, 42% reached a favorable and 29% expired. 6.5% of the patients died within the first 7 days of monitoring (median day 4, IQR 2.5–5). The distributions of available data of the cohort can be found in Supplement A. Average monitoring values were median (IQR) were 11 mmHg (7–13) for ICP, 74 mmHg (71–77) for CPP, 84 mmHg (80–89) for ABP, 74 beats per minute (71–77) for HR, and 0.00 (− 0.10–0.15) for PRx.

**Table 1:** Patient characteristics*

	N = 232
Sex (male)	176 (76%)
Age	48 (32, 60)
GCS < 8	153 (66%)
GCS Total	6 (3, 10)
GCS Eye	1 (1, 3)
GCS Verbal	2 (1, 3)
GCS Motor	4 (1, 5)
Pupillary Reactivity	
Both reactive One reactive None reactive	157 (67%) 35 (15%) 41 (18%)
Intracranial Injury	
EDH ICH SDH Contusion SAH	33 (14%) 49 (21%) 160 (69%) 139 (60%) 152 (65%)
Isolated TBI	54 (23%)
Extracranial Injury	
Abdomen Extremities Pelvis Skull Spine Thorax	34 (15%) 42 (18%) 25 (11%) 128 (55%) 49 (42%) 98 (42%)
Decompressive Craniectomy	68 (29%)

^*^shown as n (%) or median (IQR)

### Overall averages, dose, and ptime

**Fig. 2:**
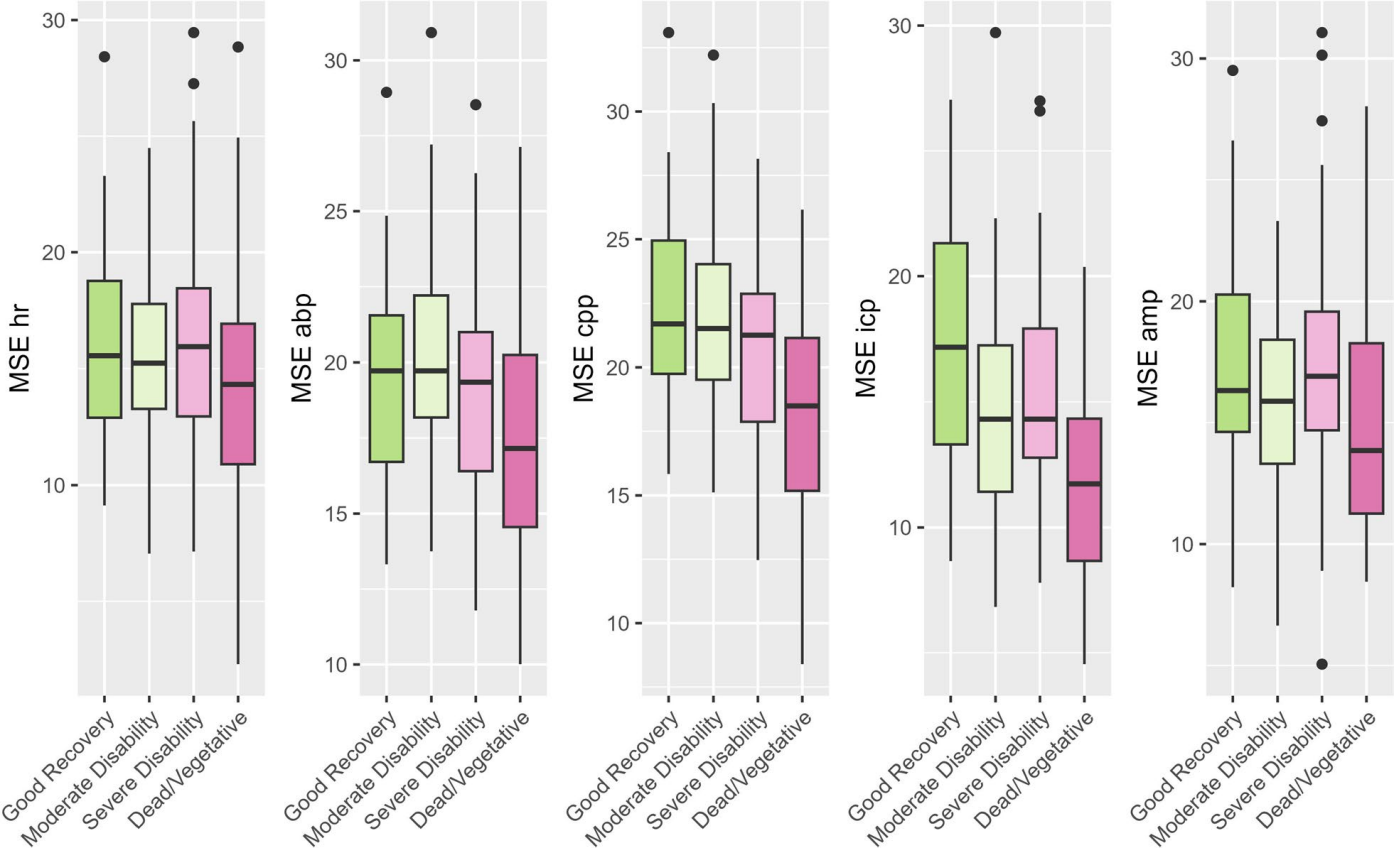
Average MSE values vs. Outcome* Average MSE values were explored using boxplots and tested using Kruskal–Wallis tests. Overall differences could be found for MSE abp (p = 0.001), MSE cpp (*p* < 0.001), MSE icp (*p* < 0.001) and MSE amp (p = 0.011) but not MSE hr (p = 0.10). The subgroup analysis can be found in Supplement B. *Abbreviations: ABP – Arterial Blood Pressure; AMP – intracranial pressure amplitude; CPP – cerebral perfusion pressure; HR – heart rate;

**Fig. 3:**
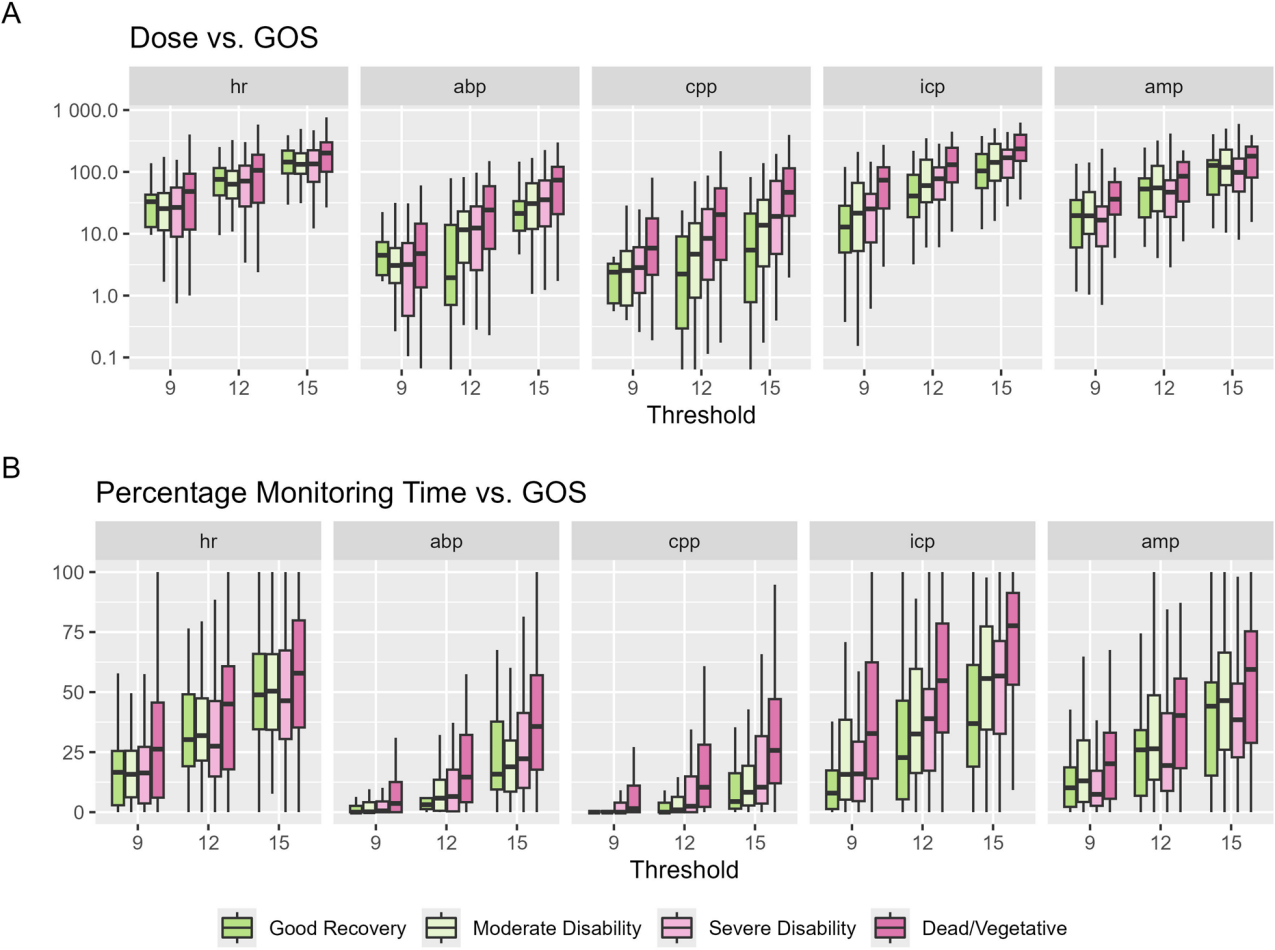
Ptime and dose below prespecified MSE thresholds for the different biosignals compared to GOS.* *Abbreviations: ABP – Arterial Blood Pressure; AMP – intracranial pressure amplitude; CPP – cerebral perfusion pressure; HR – heart rate; GOS – Glasgow Outcome Scale; MSE – Multiscale Entropy; ptime – percentage monitoring time

Overall averages of MSE aided differentiation between the different outcome groups (Fig. 2 and Supplement B) on a group level. However, within the subgroup analyses most overall MSE metrics were only different when comparing the better outcome groups vs. dead/vegetative. The other three groups could not be distinguished. Three cutoffs (i.e. MSE 9, 12, 15) were chosen based on the hourly values of MSE (Supplement C) showing an increase in proportion of lower MSE values for patients with worse outcome below ~ 15. Ptime and dose below these specific thresholds depending on GOS are shown in Fig. 3. The results of the univariable analysis considering the different cutoffs are shown in Supplement D. In comparison to average MSE values, dose and ptime below specific cutoffs of MSE icp and cpp could distinguish between other subgroups (MSE icp: good recovery vs. moderate disability and a trend when considering good recovery vs. severe disability; MSE cpp good recovery vs. severe disability) in addition to the difference between the higher groups and dead/vegetative. Considering the similar results when comparing the different cutoffs, the additional analyses were performed with the cutoff of MSE 12. The diagnostic performance of the singular metrics (ICP, CPP, PRx, and the MSE metrics) was assessed by assessing their receiver operating curves. AUCs were highest for the MSE metrics derived from ABP, CPP and ICP (AUC between 0.61–0.68). Considerably lower AUCs were derived from mean ICP (AUC 0.54) and CPP (0.49). The other measures are described in Supplement D.

### Multivariable analyses

Due to the large number of known risk-factors for unfavorable outcome in TBI, different multivariable analyses were performed. First, propensity score matching was performed to adjust the differences between the various clinical parameters (i.e. age, sex, motor GCS, pupillary reactivity, type of hemorrhage – EDH/ICH/SDH/contusion/SAH, isolated TBI vs. polytrauma, extracranial injury (to the abdomen, extremities, pelvis, skull, spine, or thorax), and DC) and outcome. A total of 122 patients were matched without relevant difference in confounders distribution post matching (Supplement E). Dose and ptime below the cutoffs were then added with ICP, CPP, and PRx to multivariable logistic regression models to assess the relevance of the metrics compared to other MMM metrics (Table 2). Only MSE abp and MSE cpp dose and ptime remained significant predictors of outcome (ptime: MSE abp—OR 1.28 (1.07–1.58) p = 0.013, MSE cpp—OR 1.50 (1.16–2.03) p = 0.005; dose: MSE abp OR 1.12 (1.02–1.27) p = 0.043, MSE cpp—OR 1.21 (1.06–1.46) p = 0.018). When considering the additional metrics (PRx, ICP, and CPP), only PRx was an additional driver of outcome after propensity score matching. Second, a sliding dichotomy approach was used to adjust the outcome definition to the initial clinical severity and then added with the different dose and ptime metrics to logistic regression models (Table 2). Similar to the propensity score approach, MSE cpp and MSE abp, but also MSE icp remained relevant predictors of outcome after TBI (ptime: MSE abp—OR 1.12 (1.02–1.24) p = 0.021, MSE cpp – OR 1.19 (1.06–1.36) p = 0.005, MSE icp – OR 1.07 (1.02–1.12) p = 0.008; dose: MSE abp – OR 1.06 (1.01–1.13) p = 0.033, MSE cpp – OR 1.09 (1.02–1.17) p = 0.015, MSE icp – OR 1.03 (1.01–1.04) p = 0.002).

**Table 2:** Multivariable analysis MSE dose and ptime vs. outcome* The different MSE metrics were compared using two ordinal multivariable methods: Propensity score matching and Sliding Dichotomy. Propensity scores were calculated (incl. age, sex, motor GCS, pupillary reactivity, type of hemorrhage – EDH/ICH/SDH/contusion/SAH, isolated TBI vs. polytrauma, extracranial injury (to the abdomen, extremities, pelvis, skull, spine, or thorax seperately), and DC) for each patient and used for matching patients with favorable and unfavorable outcome but similar presentation or for adjusting the outcome definition based on the initial severity

	Variable	Odds ratio (95% confidence interval)	p-value
Propensity Score Matching	MSE hr dose below 12	1.02 (1.00–1.05)	0.073
	MSE abp dose below 12	1.12 (1.02–1.27)	0.043
	MSE cpp dose below 12	1.21 (1.06–1.46)	0.018
	MSE icp dose below 12	1.02 (1.00–1.05)	0.12
	MSE amp dose below 12	1.01 (0.98–1.04)	0.5
	MSE hr ptime below 12	1.06 (0.97–1.16)	0.2
	MSE abp ptime below 12	1.28 (1.07–1.58)	0.013
	MSE cpp ptime below 12	1.50 (1.16–2.03)	0.005
	MSE icp ptime below 12	1.05 (0.97–1.13)	0.2
	MSE amp ptime below 12	1.02 (0.94–1.10)	0.6
Sliding Dichotomy	MSE hr dose below 12	1.01 (1.00–1.03)	0.2
	MSE abp dose below 12	1.06 (1.01–1.13)	0.033
	MSE cpp dose below 12	1.09 (1.02–1.17)	0.015
	MSE icp dose below 12	1.03 (1.01–1.04)	0.002
	MSE amp dose below 12	1.02 (1.00–1.04)	0.067
	MSE hr ptime below 12	1.02 (0.96–1.07)	0.6
	MSE abp ptime below 12	1.12 (1.02–1.24)	0.021
	MSE cpp ptime below 12	1.19 (1.06–1.36)	0.005
	MSE icp ptime below 12	1.07 (1.02–1.12)	0.008
	MSE amp ptime below 12	1.05 (1.00–1.12)	0.066

### Secondary analyses

To further explore the value of the MSE metrics compared to the known prognostic parameters associated with outcome 3 additional analyses were performed and are described in detail in Supplement F. 1. A second multivariable propensity score matching based approach was explored including ICP, CPP, and PRx doses as compared to averages. In line with the primary analysis presented in Table 2, dose and ptime of MSE abp and cpp were found to be independently associated with outcome; 2. A backwards stepwise elimination regression was applied to allow for automated simplification of the model, retaining only the key predictors of outcome. Like the previous analyses, dose and ptime of MSE abp and cpp were retained within the models after simplification; 3. To provide a quantification of improvement of the models when including the MSE metrics, we also explored the continuous net reclassification index. For this purpose, models were compared to assess the improvement of classification when including one of the MSE metrics. Moderate improvements (net reclassification index > 0.2) were found with the inclusion of MSE cpp dose and ptime as well as MSE abp ptime.

### Time trends

**Fig. 4:**
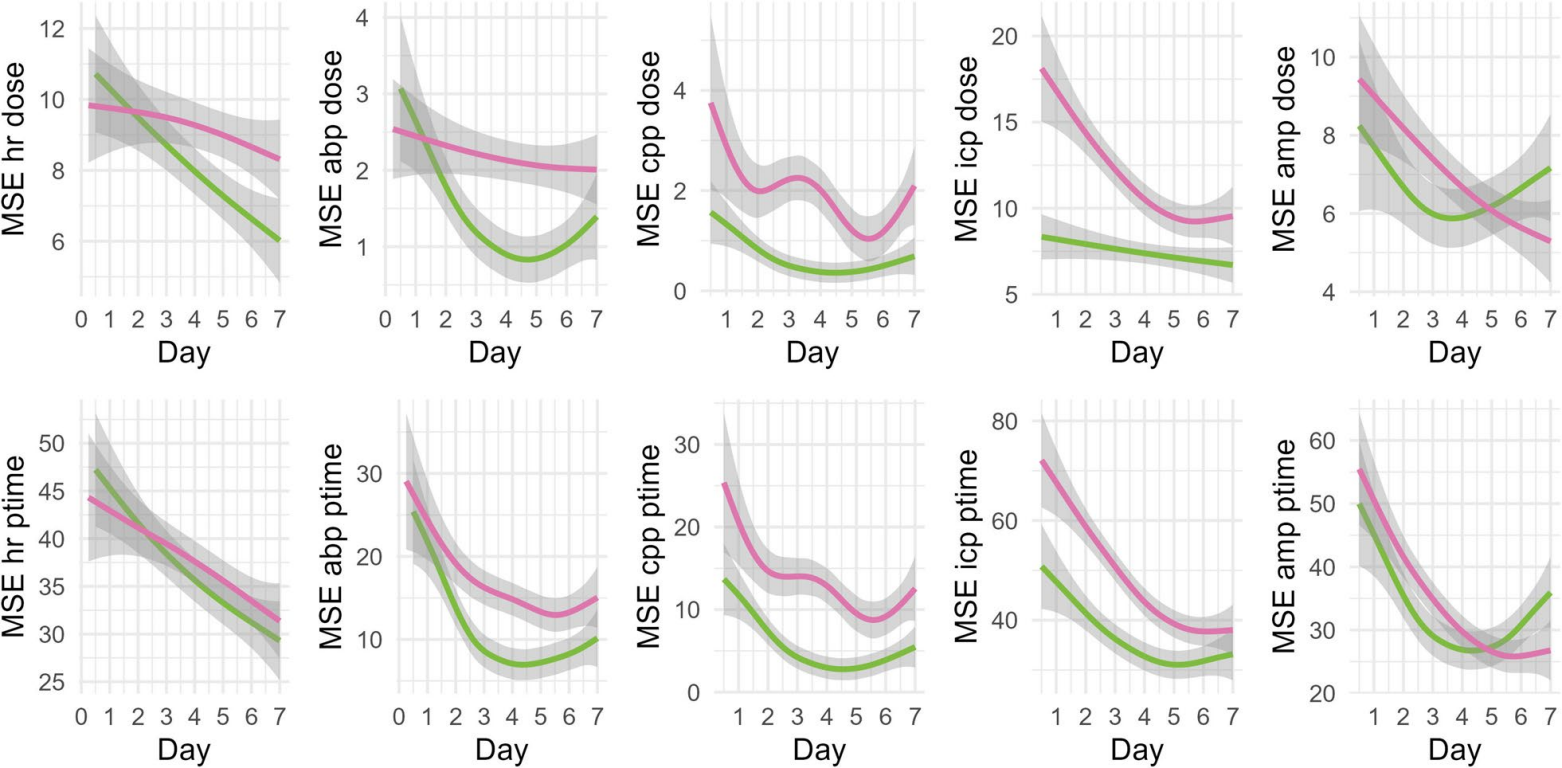
Time trends of MSE dose and ptime below a threshold depending on outcome.* MSE dose and ptime are shown as a function of time and evaluated using generalized additive models comparing MSE trajectories depending on favorable (green) and unfavorable (pink) outcome. *Abbreviations: ABP – Arterial Blood Pressure; AMP – intracranial pressure amplitude; CPP – cerebral perfusion pressure; HR – heart rate; MSE – Multiscale Entropy; ptime – percentage monitoring time

To assess a possible difference in trajectory of MSE depending on the outcome, 6 h non-overlapping averages of dose and ptime below MSE 12 of the different MSE metrics were extracted and assessed visually using GAM models (Fig. 4). Upon visual inspection, irrespective of the outcome, MSE dose and ptime showed a decrease over time. This decrease was more pronounced in patients with favorable outcome. To assess the visual differences found in the plots, the MSE metrics were added to mixed effects models assessing the trajectory of the MSE metrics depending on time and outcome with the patient added as a random effect. Within the models, MSE abp, MSE cpp, and MSE icp all displayed a significant interaction between the trajectory and outcome (*p* < 0.001). No difference in trajectory were found for MSE hr and MSE amp. When considering only the early period (first 24 h after injury), significant differences could only be detected for MSE cpp (*p* < 0.001 for ptime and dose) and MSE icp (p = 0.006 and 0.005 for dose and ptime respectively).

### Correlation to MMM and clinical metrics

**Fig. 5:**
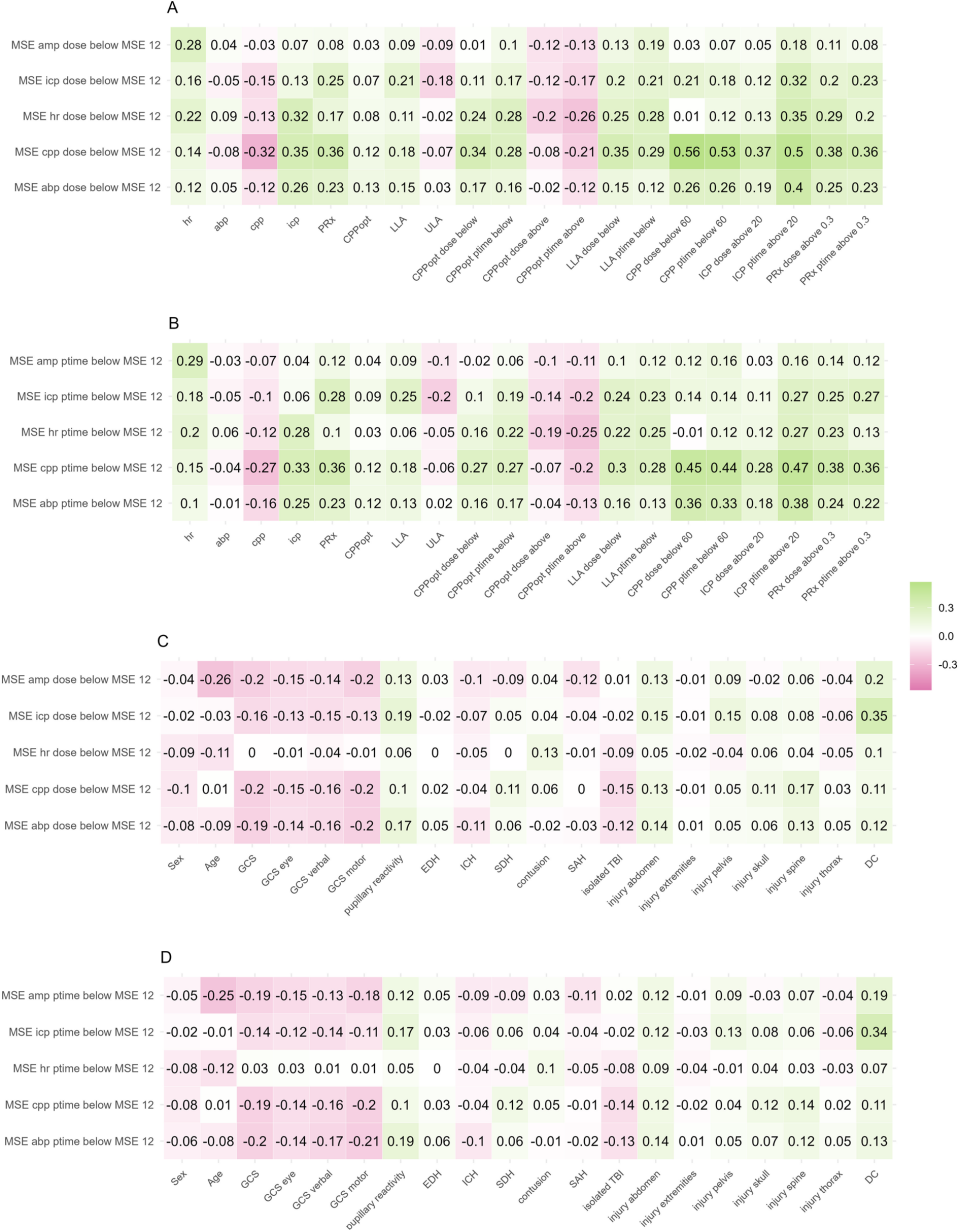
Correlation coefficients between dose or ptime of MSE below thresholds and MMM or clinical metrics. MSE of various biosignals is assessed describing dose and ptime and correlated to various monitoring and clinical metrics using pearson correlation or spearman correlation. *Abbreviations: ABP—Arterial Blood Pressure; AMP—Intracranial Pressure Amplitude; CPP—Cerebral Perfusion Pressure; CPPopt—Optimal Cerebral Perfusion Pressure; DC—Decompressive Craniectomy; EDH—Extradural Hematoma; GCS—Glasgow Coma Scale; HR—Heart Rate; ICH—Intracerebral Hemorrhage; ICP—Intracranial Pressure; LLA—Lower Limit of Autoregulation; MSE—Multiscale Entropy; Ptime—Percentage Monitoring Time; PRx—Pressure Reactivity Index; SAH—Subarachnoid Hemorrhage; SDH—Subdural Hematoma; TBI—Traumatic Brain Injury; ULA – Upper Limit of Autoregulation

Physiological connections between overall MSE metrics and overall MMM or clinical metrics were assessed by estimating the correlation coefficients (pearson or spearman rank correlation; Fig. 5). Moderate correlations were found between MSE cpp and MSE abp ptime and dose and relevant multimodal monitoring metrics such as CPP ptime/dose below 60 mmHg, ICP ptime above 20 mmHg, and PRx above 0.3. Associations to clinical metrics were overall faint with weak correlations to signs of higher TBI severity (lower GCS, worse pupillary reactivity). The only moderate correlation was found between DC and higher dose and ptime of MSE icp.

**Fig. 6:**
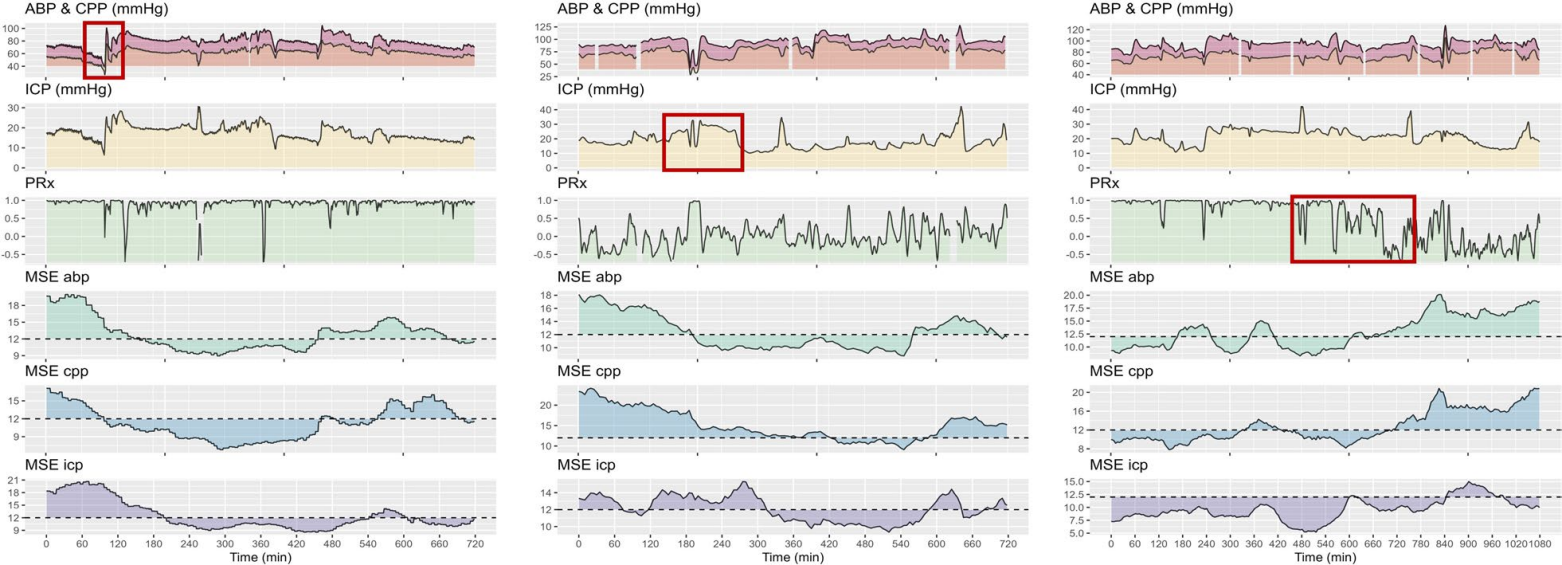
Example traces of the impact of physiological insults on MSE metrics.* This figure highlights three examples with either an episode of low cerebral perfusion pressure (left), an episode of high ICP (middle), or an extended episode of impaired cerebrovascular reactivity (right). The raw physiology metrics are shown in the top three rows. The different multiscale entropy metrics are shown in the lower three rows displaying the distance of the metric from the cutoff 12. Both occurrence of hypotension as well as intracranial hypertension led to decreases in the various MSE metrics, while an improvement of impaired cerebrovascular reactivity led to an improvement of the MSE metrics. *Abbreviations: ABP – Arterial Blood Pressure; CPP – cerebral perfusion pressure; ICP – intracranial pressure; MSE – Multiscale Entropy; PRx – Pressure Reactivity Index

Lastly, paired t-tests and mixed effects models were applied to assess the impact of individual CPP, ICP, or PRx insults on the MSE metrics using mixed effects models including the patient as a random and insult intensity/timepoint as a fixed effect. Representative examples of the dynamic changes in MSE are shown in Fig. 6. A total of 65 CPP, 78 ICP, and 198 PRx insults were identified. All different insults led to decreases of the different MSE metrics (Table 3). The temporal association between the MSE metrics which were associated with the cerebral insults were further characterized using cross-correlation. The changes in MSE metrics coincided with the onset of insult irrespective of the type of insult with a median lag of 0.

**Table 3:** Effect of individual CPP, ICP, and PRx insults on MSE.* MSE values were compared including the hour before and the hour after onset of each identified insult using either paired t-tests or linear mixed-effects models (assessing the change in MSE before vs. after the insults). The mixed effects models were adjusted for patient-specific random effects and included MSE and insult intensity (e.g., the change in CPP before vs. during the insult) as fixed effects. †denotes significant coefficients

	MSE metric	Mixed Effects Model	Paired t-test
CPP Insult	MSE hr	β −0.79 (−2.2, 0.61)	p = 0.12
	MSE abp	β −1.2 (−2.3, −0.14)†	p = 0.004
	MSE cpp	β −1.9 (−3.2, −0.52)†	p < 0.001
	MSE icp	β −0.31 (−1.8, 1.1)	p = 0.44
	MSE amp	β −0.11 (−1.3, 1.1)	p = 0.77
ICP Insult	MSE hr	β −0.58 (−1.6, 0.40)	p = 0.08
	MSE abp	β −0.86 (−1.7, −0.02)†	p = 0.003
	MSE cpp	β −1.0 (−1.9, −0.13)†	p < 0.001
	MSE icp	β −1.9 (−2.8, −1.0)†	p < 0.001
	MSE amp	β −1.2 (−2.3, −0.02)†	p = 0.002
PRx Insult	MSE hr	β −1.5 (−2.9, −0.17)†	p = 0.005
	MSE abp	β −0.74 (−1.7, 0.25)	p = 0.051
	MSE cpp	β −1.1 (−2.2, −0.03)†	p = 0.015
	MSE icp	β −1.3 (−2.3, −0.20)†	p = 0.008
	MSE amp	β −1.6 (−2.9, −0.26)†	p < 0.001

## Discussion

We describe, for the first time, the dynamic changes in biosignal entropy following TBI by assessing dose, ptime, trajectories, and their association with outcomes and relevant clinical and monitoring metrics. Our findings are consistent with previous reports, where low complexity—indicating a failure of regulating systems—was found to predict unfavorable outcomes. We validated these metrics using various statistical methods, including propensity score matching and ordinal analysis, to highlight the independent value of dynamic entropy assessment. The most important value of this study is that it offers a comprehensive analysis of the association between dynamic features of a biosignal entropy and outcome after TBI. The ICU course after TBI varies largely between patients and a substantial number of patients deteriorate during the ICU stay. Given the potential for significant changes in these patient’s conditions, it is of utmost importance to develop and implement new and improved metrics for assessing these dynamic changes. Our findings enrich the existing literature by highlighting the significance of dynamic changes in the pathophysiology of TBI and their impact on disease trajectory. Previous studies of such dynamic aspects have focused on evaluating alterations in clinical parameters such as hypoxia and pupillary changes [24], as well as laboratory biomarkers like glucose and brain-specific biomarkers [1]. Additionally, established multimodal monitoring metrics such as ICP and therapeutic intensity have been investigated [1, 25]. In contrast to static predictors of TBI outcomes, such as initial clinical severity measured by GCS or the IMPACT scores, which describe the initial severity but are unmodifiable, our research emphasizes the potential of dynamically changing metrics and disease descriptors. These dynamic parameters may be crucial for assessing the secondary trajectory of the disease and for devising strategies to prevent further secondary brain injury. By understanding and monitoring these dynamic changes, it may be possible to intervene more effectively and improve rather than just predicting long-term outcomes for TBI patients. Thus, our approach of assessing entropy dynamically might offer greater validity compared to calculating a single overall entropy value, since sample entropy, which is estimated for each MSE scale, assumes the stationarity of the system as a prerequisite. By dynamically evaluating entropy, we account for the innate non-stationary nature of the system, potentially providing more accurate and clinically relevant insights.

Considering the primary injury to be definitive, the primary focus of neurocritical care is the prevention of secondary brain injury, particularly through the management of monitoring targets such as ICP and CPP. Interestingly, our study found that MSE metrics displayed only weak associations with traditional metrics of disease severity such as pupillary response and the GCS. Our findings describing correlations between increasing ICP and decreasing MSE of different metrics corroborate previous reports by Zeiler et al.[6], who identified a similar but weaker correlation. The difference in the strength of the finding may be due to difference in methods used for entropy calculation; Zeiler assessed entropy once per patient over the first 72 h of data collection while we focused on the assessment of dynamic features of entropy throughout the first week after TBI. Conversely, and most importantly, in our study decreases in biosignal complexity derived from extracranial signals—such as ABP—showed moderate correlations with the dose and percentage of time that ICP and CPP were outside the target ranges suggested by the Brain Trauma Foundation, which is consistent with previous reports in subarachnoid haemorrhage [16]. Considering the results it is likely that the different MSE metrics represent an amalgamation of various systemic physiological processes and potentially interventions rather than a singular one.

Several explanations for the interesting associations between intracranial processes and extracranial changes in entropy are conceivable. Intracranial processes can affect the autonomic nervous system, disrupting the balance between the sympathetic and parasympathetic systems, which control cardiovascular functions including blood pressure regulation and heart rate variability [26]. Additionally, neurovascular coupling, which refers to the maintenance of adequate blood flow in response to neuronal activity, may fail after TBI. Various cardiogenic consequences of TBI including systolic dysfunction and changes in cardiac markers have been described [27]. Lastly, cardiac uncoupling after trauma increases the risk of subsequent intracranial hypertension [28]. Overall, the association between low biosignal entropy and extended periods outside the target ranges of ICP, CPP, or PRx might reflect a failure of various compensatory regulating mechanisms. The relevance of existing ICP and HR (termed “brain–heart crosstalks”) intercorrelations has previously been described and associated inversely to mortality [29]. While our results demonstrating the association between intracranial processes and extracranial signal complexity are promising, leveraging the increasing availability of high-resolution datasets, their relevance and origin need further investigation.

### Limitations

This study has several limitations including its single centre design with a moderate sample size. All of the methodologies including the MSE calculation (see below) and the propensity score based analyses [30] remain valid with the sample size and the quantity of monitoring data. The retrospective nature of the study introduces inherent biases that may affect the representativeness of the cohort and the generalizability of the findings. The cohort does not represent all consecutive patients since recordings are started by availability during the week, and dependent on the treating team on the weekends. The cohort describes a cohort of TBI patients undergoing active treatment. While the treatment of these patients is largely based on published guidelines, the exact treatment regimen is determined by the treating physician, and data on therapies that might influence variability in the parameters studied—such as vasopressors, mechanical ventilation parameters, alpha- or beta-blockers, intracranial hypertension management strategies (e.g., metabolic suppression or hypothermia), and the proportion of affected patients—are not available. It remains unknown to what extent treatment interventions may have altered the physiological metrics assessed, potentially confounding the results. It is of utmost importance to validate the results found in a more diverse multi-center cohort of patients. From a methodological perspective, for the analysis of MSE, enough data points must be included [3]. A moving window including 6 consecutive hours of data was used to calculate MSE. Thus, the calculation assessing the impact of deranged physiological parameters on the entropy metric includes some inherent overlap. MSE values calculated at the beginning of the insult considered in addition to the values of the insult some datapoints which occurred prior to the insult. While this, at an initial glance, might seem like a disadvantage, it also allows for direct evaluation of the transition from physiological values towards the insult and then the consequences after the insult. Lastly, while the changes in MSE occurred concurrently with the changes in cerebral biosignals, there are some critical aspects that merit further discussion. First, the analyses indicate that the entropy metrics demonstrated a stronger predictive value compared to ICP or CPP. Second, while no lag could be identified within the cross correlation analysis, the full complexity of temporal dynamics that may occur before the onset of an insult could not be captured. Investigating these dynamics, such as through more complex time-series analyses or predictive modeling, may uncover additional predictive value of entropy metrics. While these deeper analyses lie beyond the scope of the current manuscript, we believe the analysis of such dynamic temporal changes and the effect of interventions and medications represents a promising avenue for future research.

## Conclusions

This study provides the first analysis of time-domain features of multiscale entropy in ICU treated patients with TBI. MSE dose and ptime are independent predictors of 6-month outcome after TBI irrespective of clinical presentation, ICP, CPP, and PRx. Consistent with previous reports, there is a moderate correlation between MSE and deviations from physiological ranges of various biosignals reinforcing the notion that low MSE metrics represent failure of autoregulation systems. Of particular interest are both the novel identification of the association between signs of intracranial injury such as increased ICP or PRx and decreases in entropy derived from extracranial biosignals possibly augmenting the potential of non-invasive monitoring methods. In this context, MSE represents a potential marker of patient deterioration, which may be considered as a smart alarm, alerting the physician to an increased patient vulnerability requiring closer inspection of all the available measurements and assessments. From a clinical perspective these results, not only underline the potential of more complex metrics such as entropy for improving patient prognostication and potentially monitoring but also incentivize further exploration and exploitation of the vast datalakes acquired for these patients using bioinformatics and more advanced data-driven methods.

## Data availability statement

Postprocessed data is available upon reasonable request to the corresponding author.

## Supplementary Information

Additional file1 (DOCX 33 KB)

Additional file2 (DOCX 4037 KB)

## Abbreviations

ABP: Arterial blood pressure
AMP: Intracranial pressure amplitude
AUC: Area under the curve
CI: Confidence interval
CPP: Cerebral perfusion pressure
CPPopt: Optimal CPP
DC: Decompressive craniectomy
EDH: Extradural hematoma
GCS: Glasgow coma scale
GOS: Glasgow outcome scale
HR: Heart rate
ICH: Intracerebral hemorrhage
ICP: Intracranial pressure
IQR: Interquartile range
LLA: Lower limit of autoregulation
MSE: Multiscale entropy
NCCU: Neurocritical care unit
OR: Odds ratio
Ptime: Percentage monitoring time
PRx: Pressure reactivity index
ROC: Receiver operating characteristic
SAH: Subarachnoid hemorrhage
SDH: Subdural hematoma
TBI: Traumatic brain injury
ULA: Upper limit of autoregulation

## References

[1] Åkerlund CA, Holst A, Bhattacharyay S, Stocchetti N, Steyerberg E, Smielewski P, et al. Clinical descriptors of disease trajectories in patients with traumatic brain injury in the intensive care unit (CENTER-TBI): a multicentre observational cohort study. Lancet Neurol. 2024;23(1):71–80. 37977157 10.1016/S1474-4422(23)00358-7

[2] Muehlschlegel S, Carandang R, Ouillette C, Hall W, Anderson F, Goldberg R. Frequency and impact of intensive care unit complications on moderate-severe traumatic brain injury: early results of the Outcome Prognostication in Traumatic Brain Injury (OPTIMISM) Study. Neurocrit Care. 2013;18:318–31. 23377884 10.1007/s12028-013-9817-2

[3] Costa M, Goldberger AL, Peng C-K. Multiscale entropy analysis of biological signals. Phys Rev E. 2005;71(2): 021906. 10.1103/PhysRevE.71.021906 15783351

[4] Costa M, Goldberger AL, Peng CK. Multiscale entropy analysis of complex physiologic time series. Phys Rev Lett. 2002;89(6): 068102. 12190613 10.1103/PhysRevLett.89.068102

[5] Lu CW, Czosnyka M, Shieh JS, Smielewska A, Pickard JD, Smielewski P. Complexity of intracranial pressure correlates with outcome after traumatic brain injury. Brain. 2012;135(Pt 8):2399–408. 22734128 10.1093/brain/aws155 PMC3407422

[6] Zeiler FA, Ercole A, Placek MM, Hutchinson PJ, Stocchetti N, Czosnyka M, et al. Association between physiological signal complexity and outcomes in moderate and severe traumatic brain injury: a CENTER-TBI exploratory analysis of multi-scale entropy. J Neurotrauma. 2021;38(2):272–82. 32814492 10.1089/neu.2020.7249

[7] Riordan WP Jr, Norris PR, Jenkins JM, Morris JA Jr. Early loss of heart rate complexity predicts mortality regardless of mechanism, anatomic location, or severity of injury in 2178 trauma patients. J Surg Res. 2009;156(2):283–9. 19592027 10.1016/j.jss.2009.03.086

[8] Naraghi L, Mejaddam A, Birkhan O, Chang Y, Cropano C, Mesar T, et al. Sample entropy predicts lifesaving interventions in trauma patients with normal vital signs. J Crit Care. 2015;30(4):705–10. 25858820 10.1016/j.jcrc.2015.03.018

[9] Peev MP, Naraghi L, Chang Y, DeMoya M, Fagenholz P, Yeh D, et al. Real-time sample entropy predicts life-saving interventions after the Boston Marathon bombing. J Critical Care. 2013;28(6):1109. 10.1016/j.jcrc.2013.08.026 24120576

[10] Lu C-W, Czosnyka M, Shieh J-S, Smielewska A, Pickard JD, Smielewski P. Complexity of intracranial pressure correlates with outcome after traumatic brain injury. Brain. 2012;135(8):2399–408. 22734128 10.1093/brain/aws155 PMC3407422

[11] Vik A, Nag T, Fredriksli OA, Skandsen T, Moen KG, Schirmer-Mikalsen K, et al. Relationship of “dose” of intracranial hypertension to outcome in severe traumatic brain injury: Clinical article. J Neurosurg JNS. 2008;109(4):678–84. 10.3171/JNS/2008/109/10/0678 18826355

[12] Güiza F, Depreitere B, Piper I, Citerio G, Chambers I, Jones PA, et al. Visualizing the pressure and time burden of intracranial hypertension in adult and paediatric traumatic brain injury. Intensi Care Med. 2015;41:1067–76. 10.1007/s00134-015-3806-1 25894624

[13] Donnelly J, Czosnyka M, Adams H, Cardim D, Kolias AG, Zeiler FA, et al. Twenty-five years of intracranial pressure monitoring after severe traumatic brain injury: a retrospective, single-center analysis. Neurosurgery. 2019;85(1):E75–82. 30476233 10.1093/neuros/nyy468

[14] Menon D, Ercole A. Critical care management of traumatic brain injury. Handb Clin Neurol. 2017;140:239–74. 28187802 10.1016/B978-0-444-63600-3.00014-3

[15] Carney N, Totten AM, O’Reilly C, Ullman JS, Hawryluk GW, Bell MJ, et al. Guidelines for the management of severe traumatic brain injury. Neurosurgery. 2017;80(1):6–15. 27654000 10.1227/NEU.0000000000001432

[16] Bögli SY, Olakorede I, Veldeman M, Beqiri E, Weiss M, Schubert GA, et al. Predicting outcome after aneurysmal subarachnoid hemorrhage by exploitation of signal complexity: a prospective two-center cohort study. Crit Care. 2024;28(1):163. 38745319 10.1186/s13054-024-04939-7 PMC11092006

[17] Richman JS, Moorman JR. Physiological time-series analysis using approximate entropy and sample entropy. American journal of physiology-heart and circulatory physiology. 2000. 10.1152/ajpheart.2000.278.6.H2039 10843903

[18] Roozenbeek B, Lingsma HF, Perel P, Edwards P, Roberts I, Murray GD, et al. The added value of ordinal analysis in clinical trials: an example in traumatic brain injury. Crit Care. 2011;15:1–7. 10.1186/cc10240 PMC3218993 21586148

[19] Pencina MJ, D’Agostino RB Sr, Steyerberg EW. Extensions of net reclassification improvement calculations to measure usefulness of new biomarkers. Stat Med. 2011;30(1):11–21. 21204120 10.1002/sim.4085 PMC3341973

[20] Czosnyka M, Smielewski P, Kirkpatrick P, Laing RJ, Menon D, Pickard JD. Continuous assessment of the cerebral vasomotor reactivity in head injury. Neurosurgery. 1997;41(1):11–9. 9218290 10.1097/00006123-199707000-00005

[21] Beqiri E, Zeiler FA, Ercole A, Placek MM, Tas J, Donnelly J, et al. The lower limit of reactivity as a potential individualised cerebral perfusion pressure target in traumatic brain injury: a CENTER-TBI high-resolution sub-study analysis. Crit Care. 2023;27(1):194. 37210526 10.1186/s13054-023-04485-8 PMC10199598

[22] Beqiri E, Ercole A, Aries MJH, Placek MM, Tas J, Czosnyka M, et al. Towards autoregulation-oriented management after traumatic brain injury: increasing the reliability and stability of the CPPopt algorithm. J Clin Monit Comput. 2023;37(4):963–76. 37119323 10.1007/s10877-023-01009-1 PMC10371880

[23] Beqiri E, Czosnyka M, Placek MM, Cucciolini G, Motroni V, Smith CA, et al. Red solid line: patterns of terminal loss of cerebrovascular reactivity at the bedside. Brain and Spine. 2024;4: 102760. 38510604 10.1016/j.bas.2024.102760 PMC10951796

[24] Volpi PC, Robba C, Rota M, Vargiolu A, Citerio G. Trajectories of early secondary insults correlate to outcomes of traumatic brain injury: results from a large, single centre, observational study. BMC Emerg Med. 2018;18:1–9. 30518336 10.1186/s12873-018-0197-y PMC6280374

[25] Bhattacharyay S, Caruso PF, Åkerlund C, Wilson L, Stevens RD, Menon DK, et al. Mining the contribution of intensive care clinical course to outcome after traumatic brain injury. NPJ Digital Med. 2023;6(1):154. 10.1038/s41746-023-00895-8 PMC10442346 37604980

[26] Sykora M, Czosnyka M, Liu X, Donnelly J, Nasr N, Diedler J, et al. Autonomic impairment in severe traumatic brain injury: a multimodal neuromonitoring study. Crit Care Med. 2016;44(6):1173–81. 26968025 10.1097/CCM.0000000000001624

[27] El-Menyar A, Goyal A, Latifi R, Al-Thani H, Frishman W. Brain-heart interactions in traumatic brain injury. Cardiol Rev. 2017;25(6):279–88. 28984668 10.1097/CRD.0000000000000167

[28] Mowery NT, Norris PR, Riordan W, Jenkins JM, Williams AE, Morris JA Jr. Cardiac uncoupling and heart rate variability are associated with intracranial hypertension and mortality: a study of 145 trauma patients with continuous monitoring. J Trauma Acute Care Surg. 2008;65(3):621–7. 10.1097/TA.0b013e3181837980 18784576

[29] Dimitri GM, Beqiri E, Placek MM, Czosnyka M, Stocchetti N, Ercole A, et al. Modeling brain–heart crosstalk information in patients with traumatic brain injury. Neurocritical care. 2022:1–13. 10.1007/s12028-021-01353-7 PMC9110542 34642842

[30] Pirracchio R, Resche-Rigon M, Chevret S. Evaluation of the propensity score methods for estimating marginal odds ratios in case of small sample size. BMC Med Res Methodol. 2012;12:1–10. 22646911 10.1186/1471-2288-12-70 PMC3511219

