# Supplementary material for: Dynamic assessment of signal entropy for prognostication and secondary brain insult detection after traumatic brain injury
Source: Crit Care. 2024 Dec 30;28:436. doi: 10.1186/s13054-024-05228-z (PMC11684064; doi:10.1186/s13054-024-05228-z)
Supplement: Supplementary file 2 — Additional file2 (DOCX 4037 KB) [file 13054_2024_5228_MOESM2_ESM.docx]

# **SUPPLEMENT**

**Supplement A. Data coverage.** The data coverage is shown in form of boxplots (length - A), histograms (length per patient - B) and density (frequency of data depending on day post injury with day 0 being the day of the initial injury) depending on outcome category (C). The median number of hours available was 157 (IQR 88-279) hours for ABP/HR, and 153 (88-275) hours for ICP respectively. The vast majority was acquired within the first week of injury.


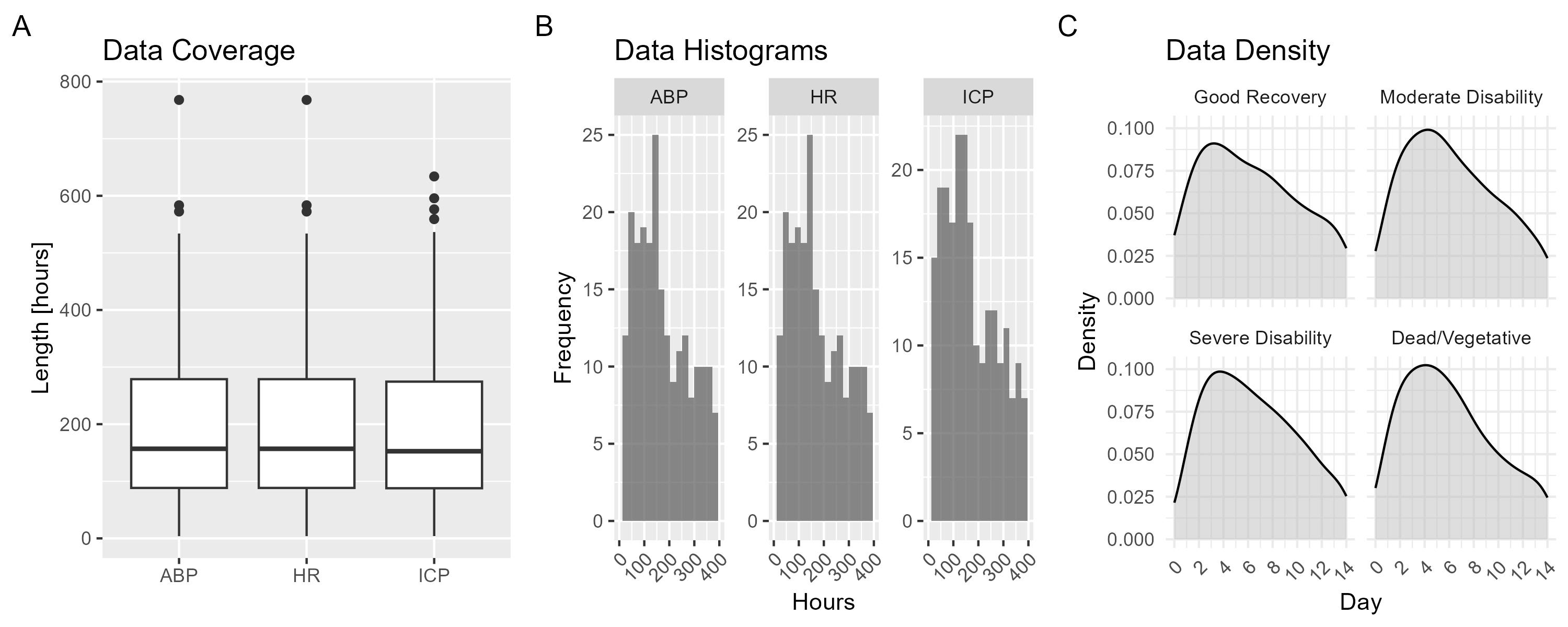


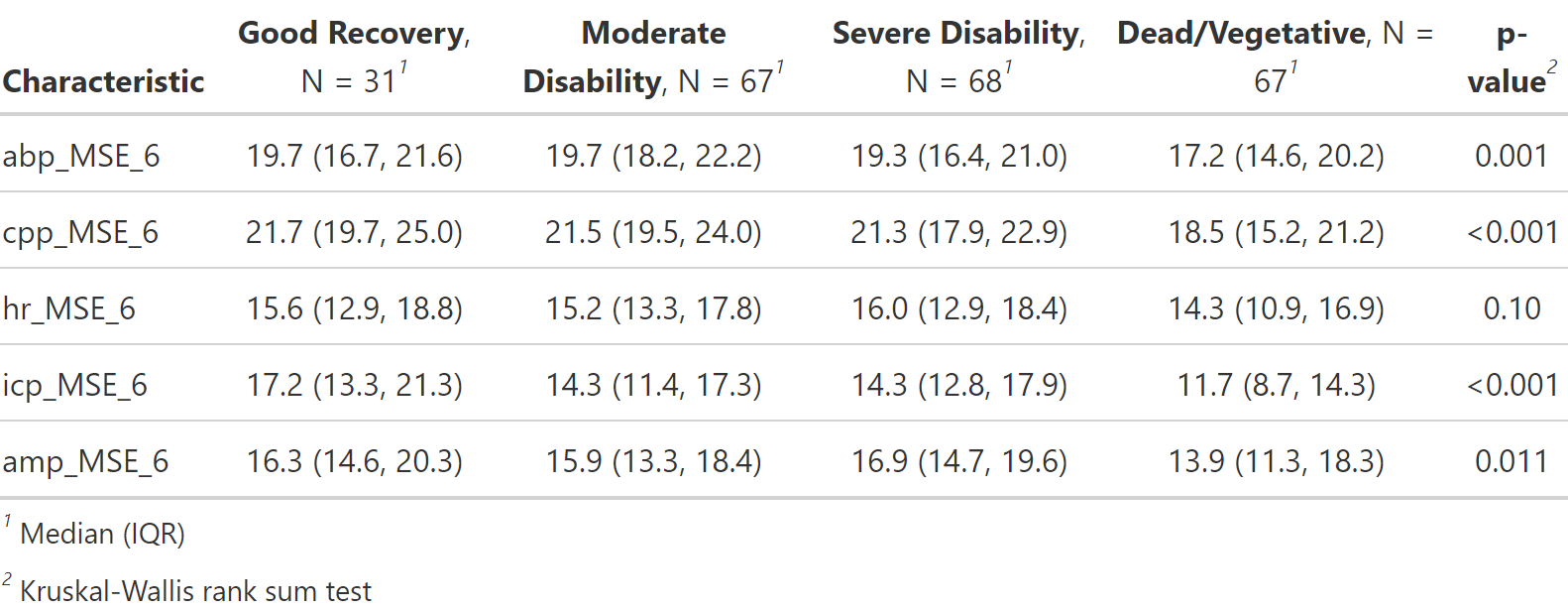
**Supplement B. Average MSE values vs. Outcome.** Average MSE values were explored using Kruskal-Wallis tests. Subgroup analysis was performed using Wilcoxon rank sum tests.


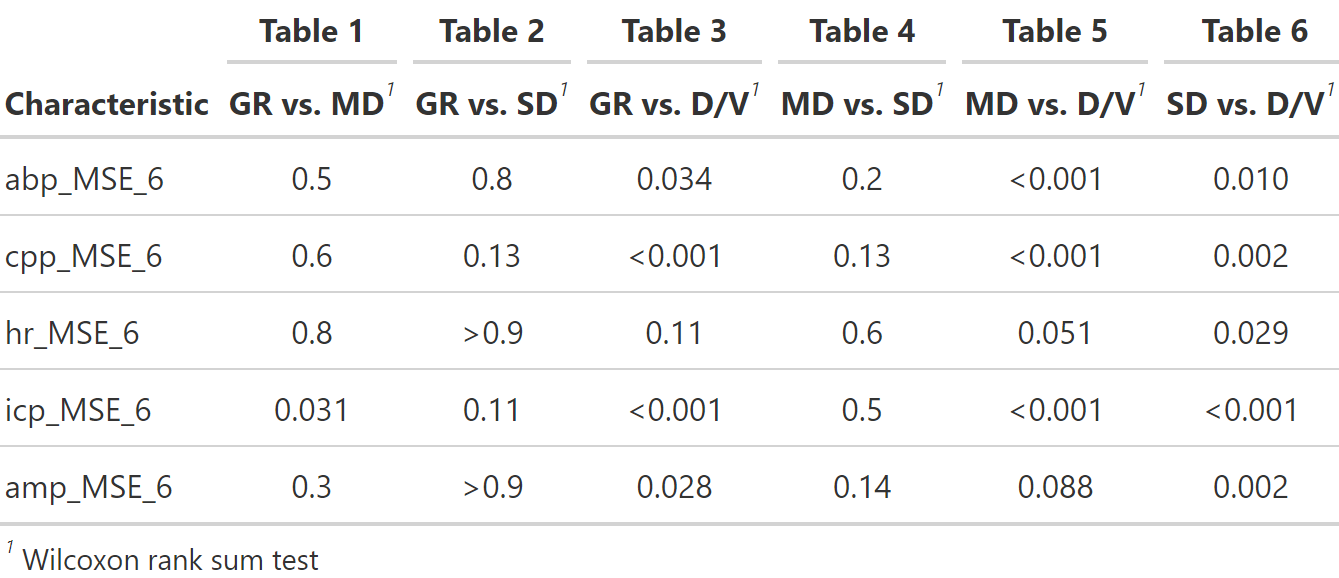


**Supplement C. Figure – Exploration hourly values of MSE.** Hourly values of MSE were extracted and plotted using stacked proportional histograms to reveal their distribution depending on ordinal outcome. A clear increase in proportion of Dead/Vegetative was found when considering MSE values around 15. Consequently, the cutoffs 15, 12, and 9 were explored assessing the dose or percentage monitoring time below this cutoff.


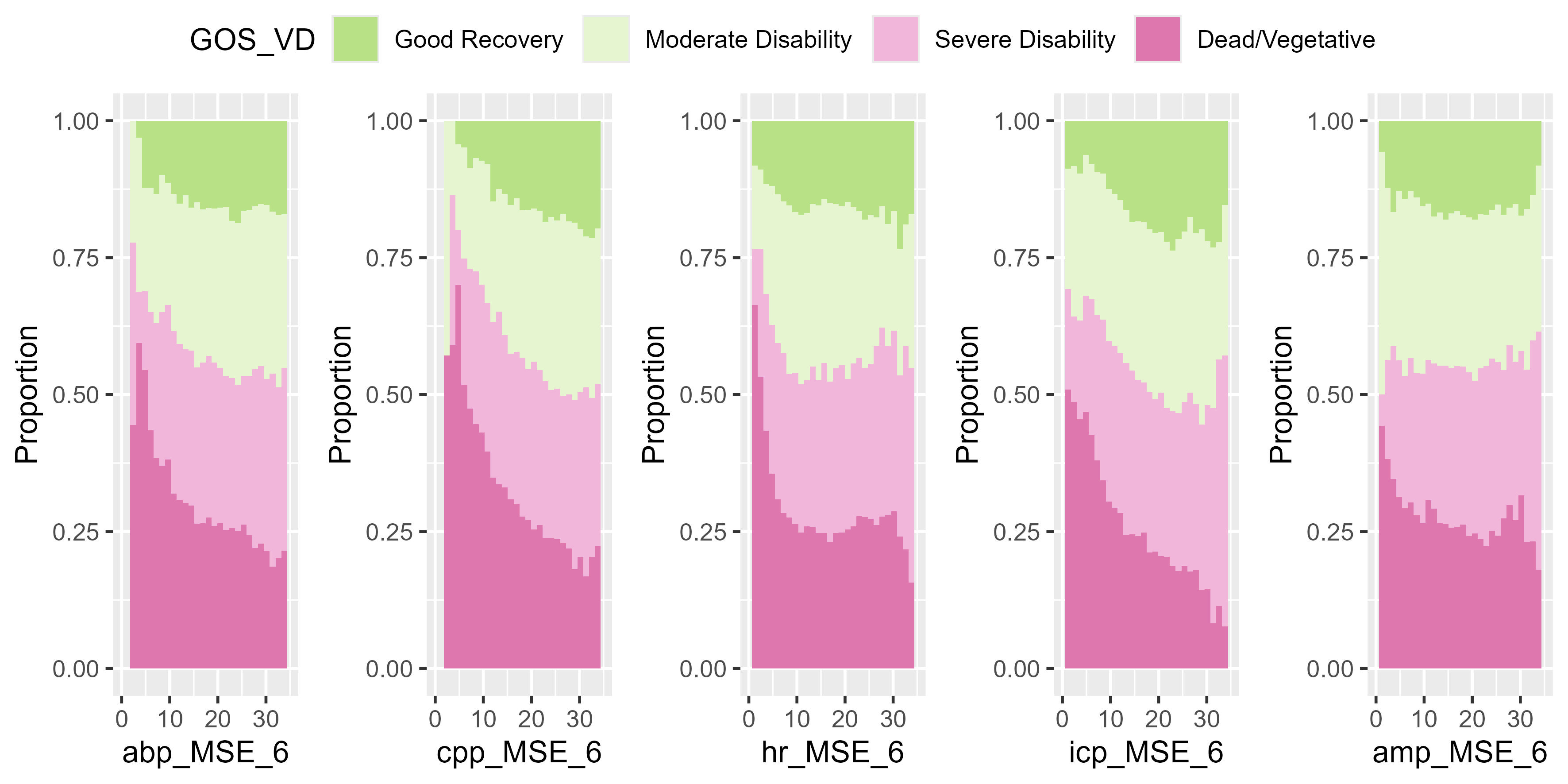


**Supplement D. Univariable analyses:**

*Dose and Ptime vs. GOS*: Dose and percentage time below different MSE cutoffs were explored using Kruskal-Wallis tests.


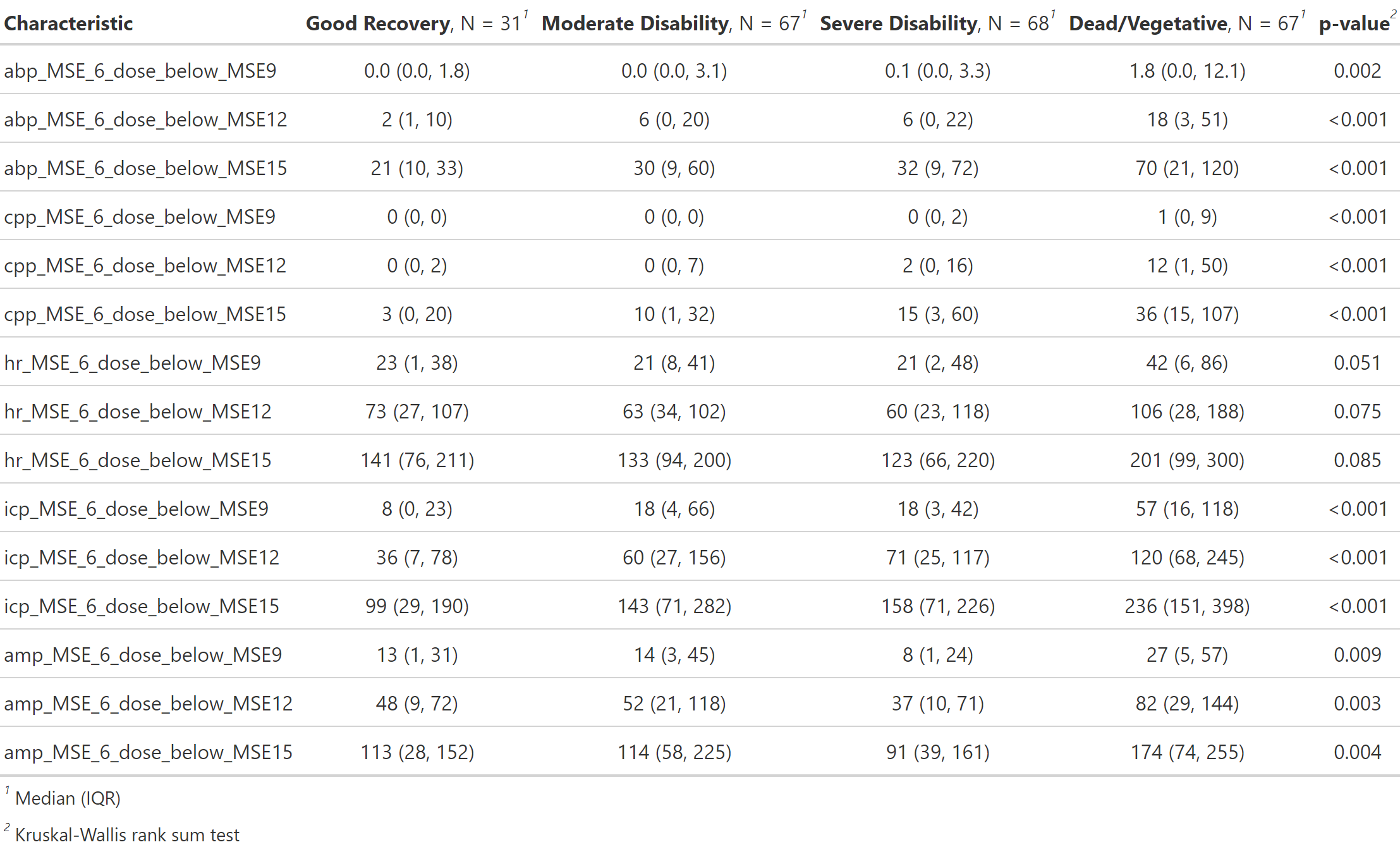

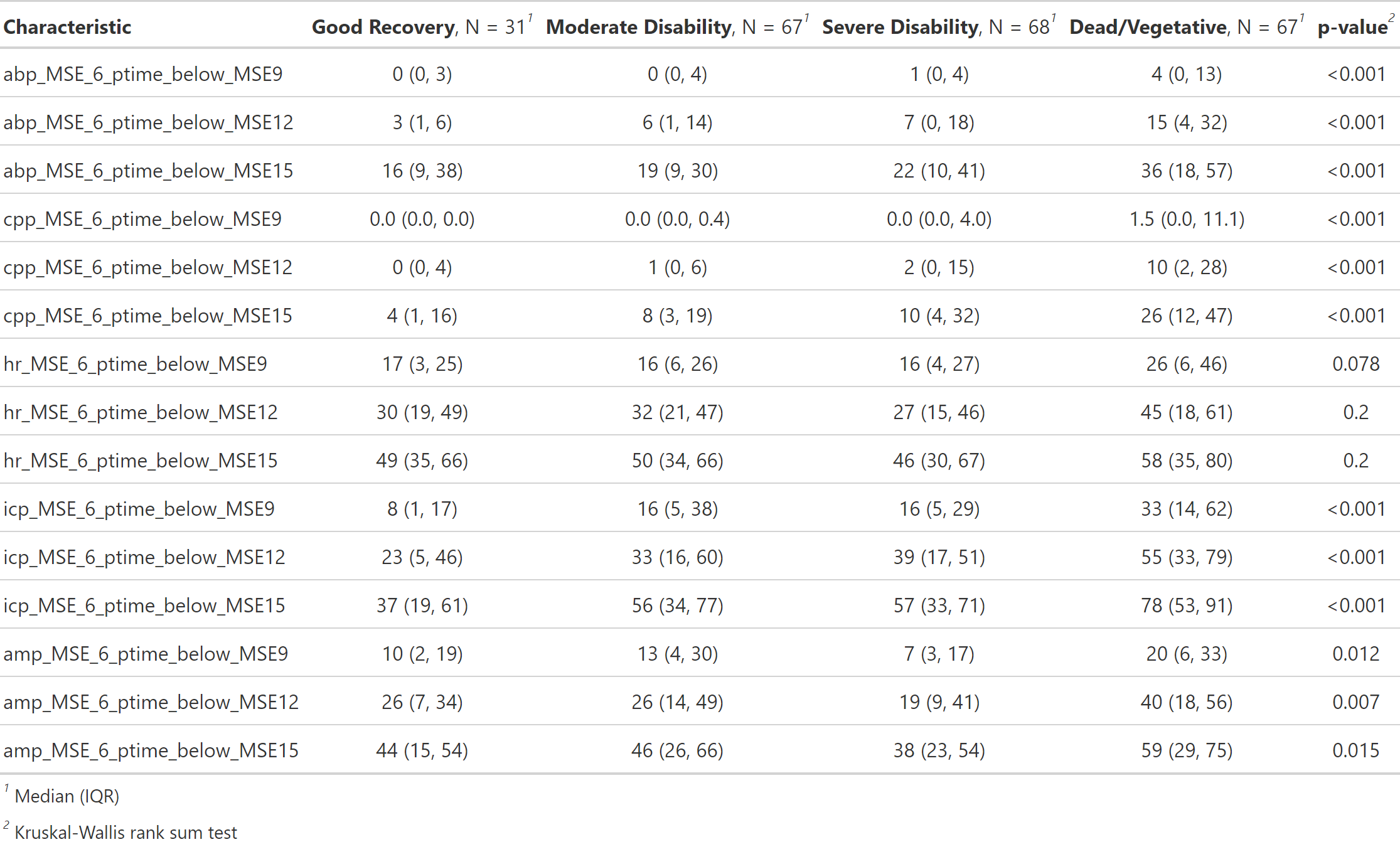


*Dose and Ptime vs. GOS - Subgroup Analysis*. Subgroup analysis was performed using Wilcoxon rank sum tests.


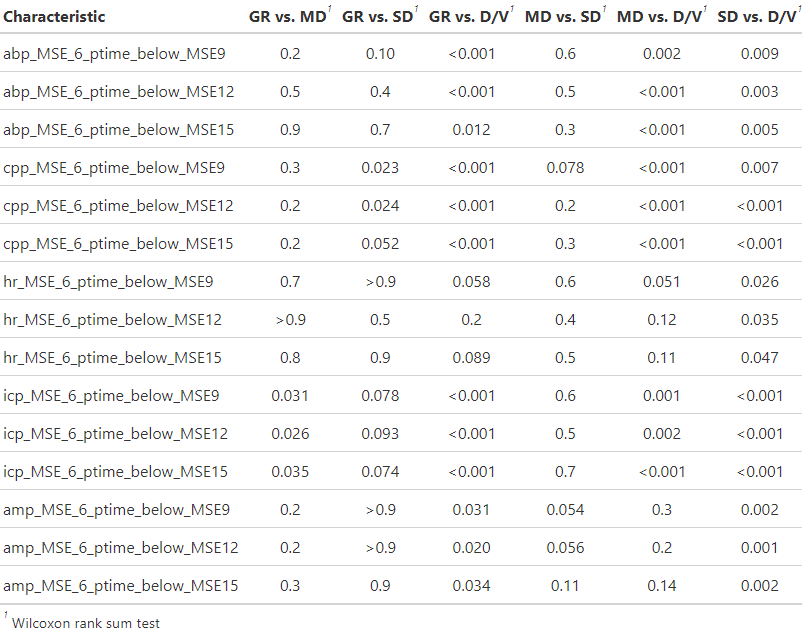

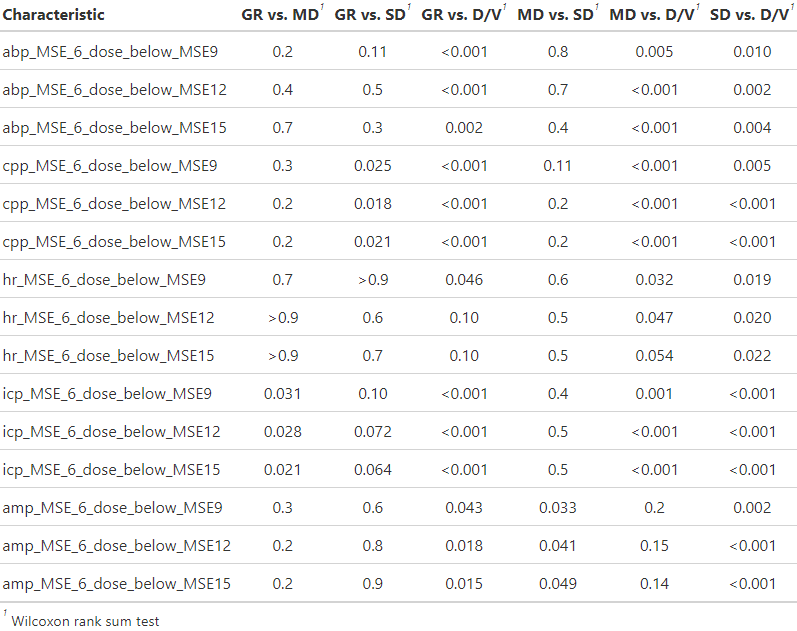


*Univariable diagnostic performance:* Diagnostic performance of the singular metrics (both known multimodality monitoring metrics and the MSE metrics) were assessed by plotting their receiver operating curves and extracting the AUC (area under the curve), sensitivity, specificity and accuracy (based on the Youden index). AUCs were highest for the MSE metrics derived from ABP and CPP and to a lesser degree ICP. In these cases, specificity was higher than sensitivity for the prediction of unfavorable outcome highlighting the benefit for correctly identifying patients with favorable outcome. The prognostic value of ICP and CPP was low in this cohort of patients.

| **Variable** | **AUC** | **Sensitivity** | **Specificity** | **Accuracy** |
| --- | --- | --- | --- | --- |
| ICP | 0.54 | 0.40 | 0.79 | 0.57 |
| CPP | 0.49 | 0.27 | 0.66 | 0.44 |
| PRx | 0.70 | 0.54 | 0.79 | 0.65 |
| MSE hr dose below 12 | 0.54 | 0.41 | 0.75 | 0.55 |
| MSE abp dose below 12 | 0.61 | 0.51 | 0.67 | 0.58 |
| MSE cpp dose below 12 | 0.67 | 0.56 | 0.75 | 0.64 |
| MSE icp dose below 12 | 0.61 | 0.64 | 0.59 | 0.62 |
| MSE amp dose below 12 | 0.51 | 0.49 | 0.59 | 0.54 |
| MSE hr ptime below 12 | 0.54 | 0.41 | 0.75 | 0.55 |
| MSE abp ptime below 12 | 0.61 | 0.51 | 0.67 | 0.58 |
| MSE cpp ptime below 12 | 0.68 | 0.53 | 0.75 | 0.62 |
| MSE icp ptime below 12 | 0.61 | 0.64 | 0.59 | 0.62 |
| MSE amp ptime below 12 | 0.51 | 0.49 | 0.59 | 0.54 |

**Supplement E. Propensity Score Matching.**

Propensity score matching was performed using the nearest-neighbour method with a caliper of 0.2 and 1:1 matching to ensure that matches were within a reasonable distance in terms of their propensity scores. The propensity scores were built using logistic regression comparing favourable vs. unfavourable outcome. The results of the matching are shown in A (density distribution of propensity scores before and after adjustment – blue: favourable outcome, red: unfavourable outcome), B (point distribution of matched and unmatched units), C (covariate balance before and after matching), and D (density plots of continuous variables before and after matching). In addition, the variables (pre and post matching) are shown in the table below.


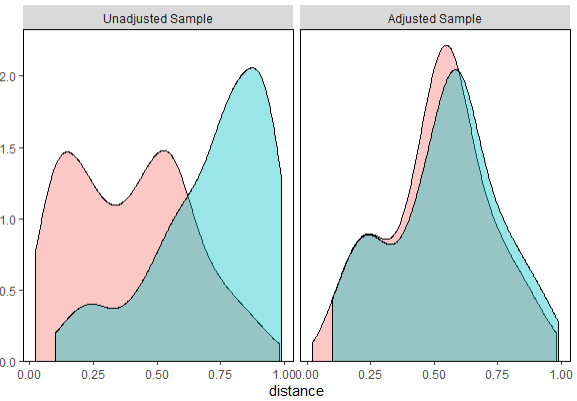

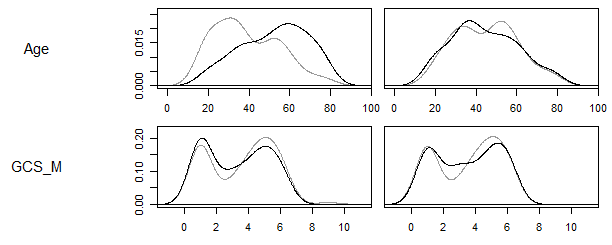


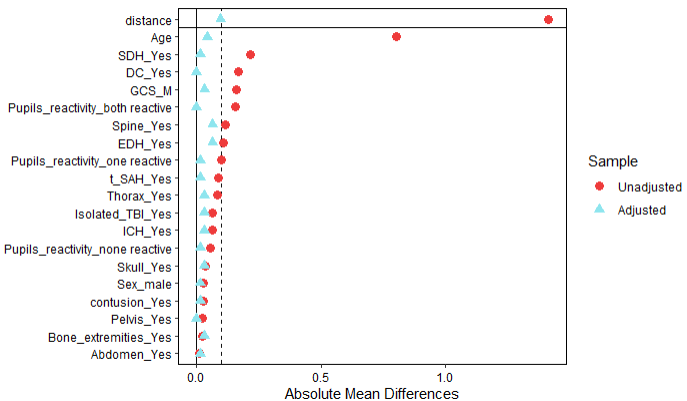

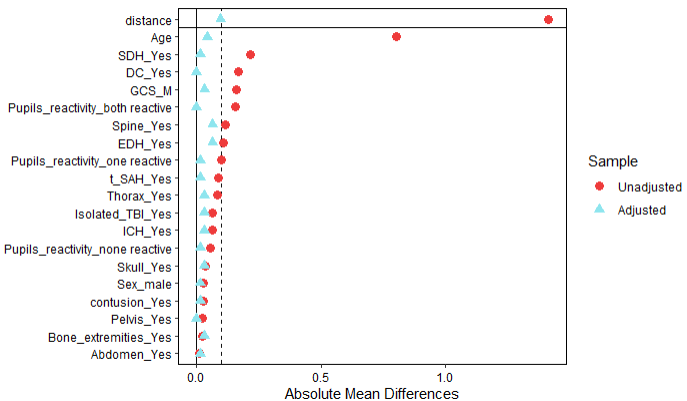

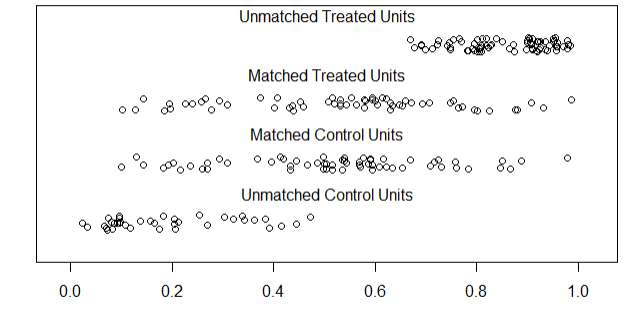

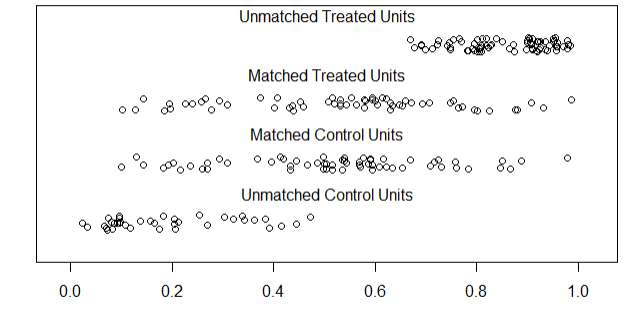


Matched: Unfavorable Outcome

Matched: Favorable Outcome

Unmatched: Favorable Outcome

Unmatched: Unfavorable Outcome


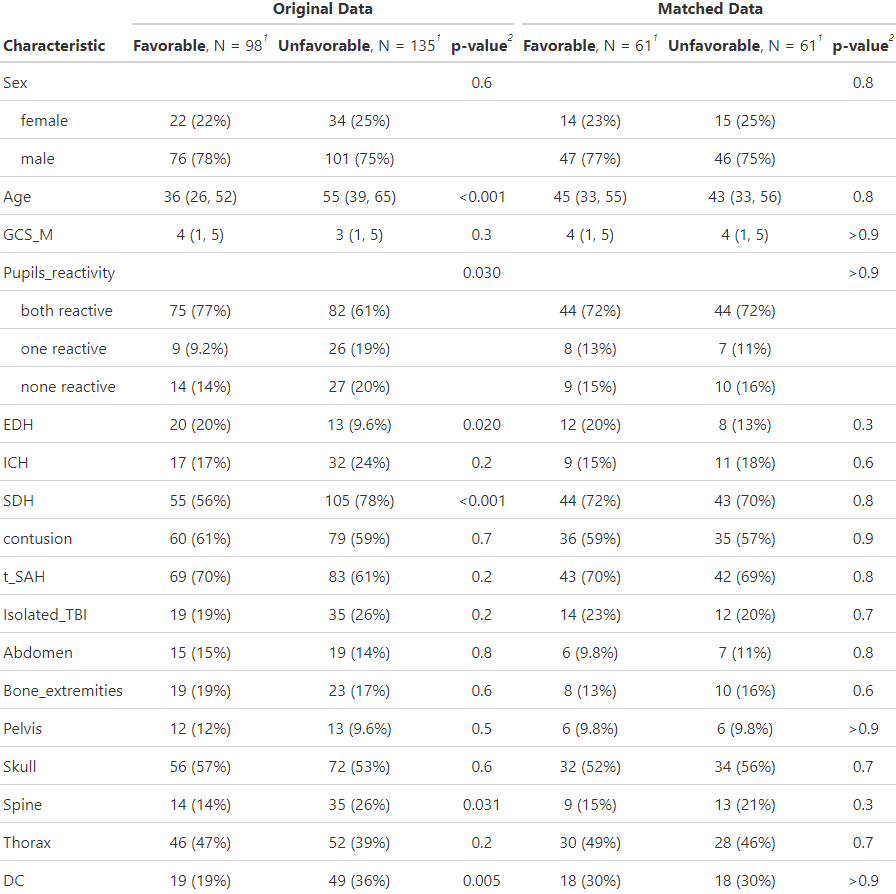


**Supplement F. Secondary statistical analyses.**

A second multivariable propensity score matching based approach was explored including ICP, CPP and PRx doses instead of averages to mimic the dynamic assessment of MSE explored in this analysis. For this purpose, patients were matched as described in the main manuscript using propensity scores (based on age, sex, motor GCS, pupillary reactivity, type of hemorrhage – EDH/ICH/SDH/contusion/SAH, isolated TBI vs. polytrauma, extracranial injury (to the abdomen, extremities, pelvis, skull, spine, or thorax separately), and DC). These matched patients were then compared using multivariable regression models including ICP, CPP and PRx doses (i.e. ICP dose above 20 mmHg, CPP dose below 60 mmHg, PRx dose above 0.3) and either MSE metric. In line with the primary analysis presented in Table 2 of the main manuscript, dose and ptime of MSE abp and cpp were found to be independently associated with outcome (see table below).

|  | **Variable** | **Odds Ratio (95% confidence interval)** | **p-value** |
| --- | --- | --- | --- |
| **Propensity Score Matching** | MSE hr dose below 12 | 1.02 (0.99-1.05) | 0.13 |
|  | MSE abp dose below 12 | **1.12 (1.01-1.28)** | **0.047** |
|  | MSE cpp dose below 12 | **1.21 (1.05-1.46)** | **0.023** |
|  | MSE icp dose below 12 | 1.02 (0.99-1.05) | 0.14 |
|  | MSE amp dose below 12 | 1.01 (0.98-1.04) | 0.7 |
|  | MSE hr ptime below 12 | 1.04 (0.95-1.14) | 0.4 |
|  | MSE abp ptime below 12 | **1.30 (1.08-1.62)** | **0.011** |
|  | MSE cpp ptime below 12 | **1.52 (1.17-2.08)** | **0.004** |
|  | MSE icp ptime below 12 | 1.05 (0.97-1.14) | 0.3 |
|  | MSE amp ptime below 12 | 1.01 (0.93-1.09) | 0.9 |

To explore further the incremental value of MSE metrics in light of the various known clinical prognostic parameters, two additional methods were explored: 1. Backwards stepwise elimination – In light of the moderate sample size with various known clinical and monitoring metrics associated with outcome, the backward stepwise elimination process was chosen since it allows for automated simplification of the model, retaining only the key predictors of outcome (R library *MASS*). The initial model was built including the various clinical metrics (age, sex, motor GCS, pupillary reactivity, type of hemorrhage – EDH/ICH/SDH/contusion/SAH, isolated TBI vs. polytrauma, extracranial injury (to the abdomen, extremities, pelvis, skull, spine, or thorax seperately), DC), the multimodality monitoring metrics (ICP, CPP, PRx) and either MSE metric. The model was then fed through a backward stepwise elimination process to identify the relevant clinical predictors of outcome (see table below). In line with the other methods, dose and ptime of MSE abp and cpp were retained within the models. 2. To provide a quantification of improvement of the models when including the MSE metrics, we also explored the continuous net reclassification index (R library *Hmisc*). Considering the number of patients the initial model included ICP dose above 20 mmHg, CPP dose below 60 mmHg, and PRx. This model was then compared to a model including the described metrics as well as one of the MSE metrics. The continuous net reclassification index represents the proportions of individuals correctly reclassified (true positive or true negative), minus the proportion misclassified when comparing the new to the old models.^1^ Moderate improvements (index >0.2) were found with the inclusion of MSE CPP dose and ptime as well as MSE abp ptime.

|  | **Backward stepwise elimination** | | **Net reclassification index** |
| --- | --- | --- | --- |
| **Variable** | **Odds Ratio (95% confidence interval)** | **p-value*** |  |
| MSE hr dose below 12 | NA | NA | 0.04 |
| MSE abp dose below 12 | **1.10 (1.02-1.19)** | **0.024** | 0.14 |
| MSE cpp dose below 12 | **1.18 (1.06-1.36)** | **0.007** | 0.36 |
| MSE icp dose below 12 | NA | NA | 0.17 |
| MSE amp dose below 12 | NA | NA | 0.05 |
| MSE hr ptime below 12 | NA | NA | 0.08 |
| MSE abp ptime below 12 | **1.10 (1.02-1.19)** | **0.024** | 0.32 |
| MSE cpp ptime below 12 | **1.18 (1.06-1.36)** | **0.007** | 0.37 |
| MSE icp ptime below 12 | NA | NA | 0.22 |
| MSE amp ptime below 12 | NA | NA | 0.00 |

^1^Pencina, M. J., D'Agostino Sr, R. B., & Steyerberg, E. W. (2011). Extensions of net reclassification improvement calculations to measure usefulness of new biomarkers. Statistics in medicine, 30(1), 11-21.
